# Recent Advances in the Synthesis of Polymer-Grafted Low-K and High-K Nanoparticles for Dielectric and Electronic Applications

**DOI:** 10.3390/molecules26102942

**Published:** 2021-05-15

**Authors:** Bhausaheb V. Tawade, Ikeoluwa E. Apata, Nihar Pradhan, Alamgir Karim, Dharmaraj Raghavan

**Affiliations:** 1 Department of Chemistry, Howard University, Washington, DC 20059, USA; bhausaheb.tawade@howard.edu (B.V.T.); ikeoluwa.apata@bison.howard.edu (I.E.A.); 2 Department of Chemistry, Physics and Atmospheric Science, Jackson State University, Jackson, MS 39217, USA; nihar.r.pradhan@jsums.edu; 3 Department of Chemical and Biomolecular Engineering, University of Houston, Houston, TX 77204, USA; akarim3@central.UH.edu

**Keywords:** polymer-grafted nanoparticles, dielectric properties, energy density, SiO_2_, TiO_2_, BaTiO_3_, Al_2_O_3_, reversible deactivation radical polymerization, ATRP, RAFT, NMP, click chemistry

## Abstract

The synthesis of polymer-grafted nanoparticles (PGNPs) or hairy nanoparticles (HNPs) by tethering of polymer chains to the surface of nanoparticles is an important technique to obtain nanostructured hybrid materials that have been widely used in the formulation of advanced polymer nanocomposites. Ceramic-based polymer nanocomposites integrate key attributes of polymer and ceramic nanomaterial to improve the dielectric properties such as breakdown strength, energy density and dielectric loss. This review describes the “grafting from” and “grafting to” approaches commonly adopted to graft polymer chains on NPs pertaining to nano-dielectrics. The article also covers various surface initiated controlled radical polymerization techniques, along with templated approaches for grafting of polymer chains onto SiO_2_, TiO_2_, BaTiO_3_, and Al_2_O_3_ nanomaterials. As a look towards applications, an outlook on high-performance polymer nanocomposite capacitors for the design of high energy density pulsed power thin-film capacitors is also presented.

## 1. Introduction

**Figure 1:**
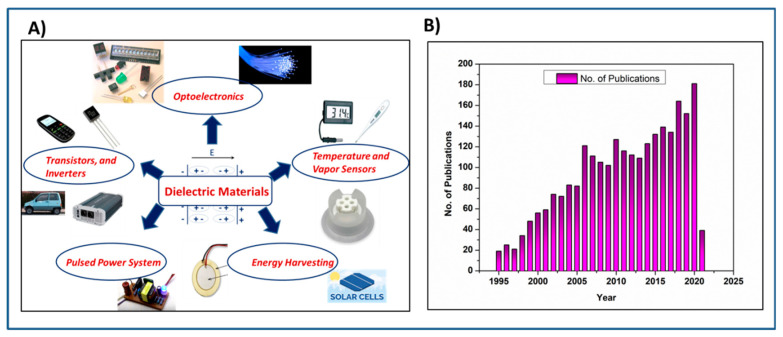
(**A**) The emerging applications of dielectric capacitors (**B**) Number of publications on “polymer dielectric capacitor” per year in the last 25 years.

The growing demand for power electronics and energy storage serves as an excellent motivation for developing next generation dielectrics and electrical insulation materials [1,2]. Dielectric polymers and polymer nanocomposites stand out as next generation dielectric materials for many electrical insulation and energy storage applications owing to their high dielectric strength, high voltage endurance, low dielectric loss, low equivalent series resistance, a gradual failure mechanism, light weight, low cost and ease of processability [3,4,5,6,7,8,9,10]. The use of polymer-based dielectric capacitors in various sectors is summarized in Figure 1A. As a result of numerous emerging potential applications of polymer-based dielectric materials and capacitors, research on strategies for enhancing capacitive energy storage methods has experienced significant growth. Figure 1B shows the number of yearly publications in the last 25 years on the topic of “dielectric polymer capacitor” as found in the Sci-Finder database. Clearly, over the years the research interest in the field of polymer dielectric capacitors has grown exponentially. 

Apart from the use of polymers in nanocomposites, inorganic materials such as ceramics are critical components for nanocomposite capacitors due to their extremely large dielectric constants, often times >1000. Despite their high dielectric constants, inorganic materials suffer from a low breakdown strength and non-graceful failure mode. Polymer nanocomposites integrate key attributes of polymer and ceramic nanomaterial to improve the overall dielectric properties [11,12].

Several comprehensive review articles including a couple of review articles from our group have been published in the field of polymer and polymer nanocomposite dielectrics [13,14,15,16,17,18,19,20,21,22,23,24,25,26]. Our first review article dealt with coverage of the nanoscale strategies in the field of polymeric and polymer nanocomposites for use in emerging dielectric capacitor-based energy storage applications [13]. Some of the strategies to address permittivity contrast between nanofillers and the polymer matrix including potential for developing gradient permittivity structured nanofillers were presented. Additionally, we had described approaches to improve the compatibility of nanofiller with polymer, minimize nanofiller aggregation, and mitigate the permittivity contrast between nanofiller and polymer, In our second review article, we discussed different chemical routes for surface functionalization of ceramic nanoparticles [14]. For instance, the article dealt with the synthesis of low-k and high-κ nanomaterials [19,20,21,22,23,24] as well as surface functionalization of nanomaterials including treatment with hydrogen peroxide, silane coupling agents, phosphonic acid and dopamine moieties that improved the interaction between nanomaterials and polymer matrix.

In the review article published in *Nanotechnology* [14], it was pointed out that the selection of the surface modifying coupling agent on the surface of nanoparticles/layer dictate the dielectric properties of the nanocomposites as well as the performance of the bilayer as it relates to gate dielectrics. Although, functionalization of nanomaterials with chemical agents is less cumbersome and less equipment intensive there are several shortcomings to adopting this method viz., (i) the structure of the chemical modifying agent is distinctly different from the long chain of polymer matrix (ii) side reaction of the chemical agent could lead to multilayer formation and (iii) physical adsorption of the modifying agent. Unlike the surface modification of nanoparticles with chemical agents, the polymer grafting of the nanoparticles yield nanoparticles with surface energy which closely matches with that of the polymer matrix. The improved compatibility of polymer-grafted nanoparticles with polymer matrix often yields nanocomposites with superior properties compared to nanocomposites with chemical agent-modified nanoparticles. For instance, maximum energy density and extraction efficiency values for polymethylmethacrylate (PMMA) grafted BaTiO_3_ filled PMMA nanocomposites was found to be two fold higher than that of coupling agent surface-modified BaTiO_3_ filled PMMA nanocomposites [27]. 

There are many approaches to improve the compatibility of the nanoparticles with polymer matrix, the nanoparticles spatial dispersion in the matrix and decrease the permittivity contrast between polymer and nanoparticles. Approaches could be based on the use of by external triggers such as a simple control of the film processing conditions (controlling % loading of filler) [28] or, of the electrostatic repulsion (tuning by change pH) [29] or with a magnetic field (tuning based on magnetic field) [30] or an internal trigger such as chemical/polymer grafting approach [31,32]. This article only deals with internal trigger (by synthesis of polymer-grafted nanoparticles) to address the compatibility of nanoparticles and polymer. Several recent reviews have comprehensively covered the topic of polymer grafting of nanoparticles [33,34,35,36,37,38,39,40]. For example, the review by Ameduri et al., [35] dealt primarily with grafting of polymers on high-K NPs (BaTiO_3_) for use in the formulation of high energy storage fluorinated polymer nanocomposites. In the present review, we cover the synthesis of polymer-grafted high-K and low-K nanoparticles for the fabrication of nanocomposites for electronics and dielectric application. Unlike, Yang et al.’s [38] review which discusses only the synthesis of polymer-grafted high and low K-nanoparticles using surface initiated-polymerization approaches, our review will cover the broad gamut of approaches available to synthesize polymer-grafted silicon dioxide (SiO_2_), titanium dioxide (TiO_2_), barium titanate (BaTiO_3_), and aluminum oxide (Al_2_O_3_) nanoparticles and their applications as dielectrics and electronics.

**Figure 2:**
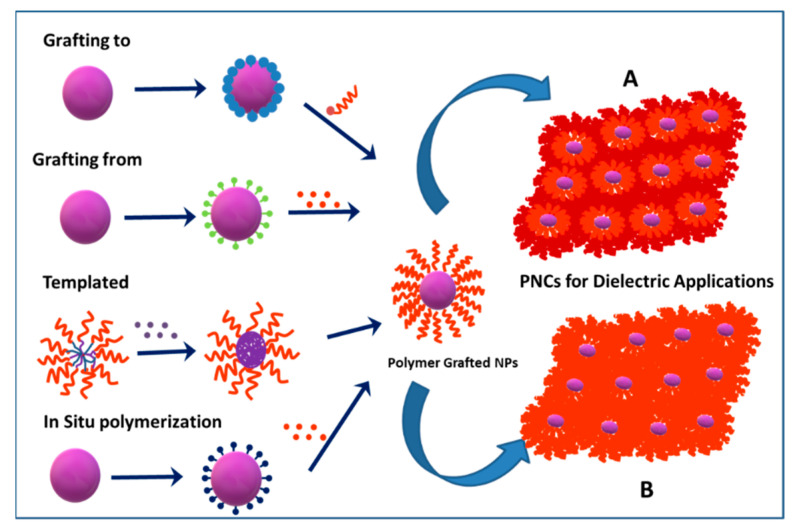
Approaches for grafting polymer chains on the surface of nanomaterials. (**A**) Polymer-nanocomposite formed from polymer-grafted NPs and polymer matrix. (**B**) Polymer nanocomposite formed from only polymer-grafted NPs).

The grafting of polymeric chains to nanoparticles can generally be achieved by four approaches namely (i) “grafting to”; (ii) “grafting from”; (iii) templated and (iv) in situ polymerization or encapsulation. Figure 2 presents pictorially the various approaches commonly adopted to prepare polymer-grafted nanoparticles. All the four approaches yield polymer-grafted nanoparticles of varying shell architecture. The polymer graft conformation on the nanoparticles is a result of the covalent bond formation that compensates for the entropy loss resulting from the polymer chains stretching away from the surface. If the polymer chains on the grafted nanoparticles have molecular weight lower than the entanglement molecular weight, then the harvested nanoparticles are commonly blended with virgin polymer to form polymer nanocomposite. On the other hand, if the molecular weight of the polymer chains on the polymer-grafted nanoparticles is far greater than the entanglement molecular weight, a nanocomposite could be formed without the addition of an external polymer matrix. The former is called multi component system while the latter is called single component system [37,41,42,43]. 

As represented in Figure 2, encapsulation or in situ polymerization approach is based on monomers being initially adsorbed on the NPs surface, and initiation of polymerization of the adsorbed monomer layer, yielding polymer-coated NPs. Sometimes the encapsulation approach could be termed as in situ grafting through approach because the monomers adsorbed on the NPs undergo polymerization in the presence of initiator in the bulk [44,45]. The second approach uses block copolymer-based micelle-template in the synthesis of hairy nanoparticles (HNPs). In this method, a precursor, commonly a metal salt or an organometallic compound, is loaded into the core of polymer micelles based on either multi-molecular block copolymer or unimolecular star block copolymer. The reduction of (complex) metal ions in the micelle core yields core–shell NPs [46,47]. The third approach is based on *grafting-to* which involves the attachment of end-functionalized polymer chains on the surface of NPs via suitable chemical reactions. A variety of reactions such as esterification, silylation, click reactions including thiol-ene, alkyne-azide cycloaddition, etc. have generally been utilized in the grafting to approach. The fourth approach is based on *grafting-from/SI-CRP* which consists of growing polymer chains directly from the surface of nanoparticles functionalized with suitable initiator/CTA functionalities. There have been remarkable developments in the surface-initiated controlled radical polymerization (SI-CRP) route for the synthesis of polymer-grafted nanoparticles [33,48,49,50,51,52]. Pioneering work from Matyjaszewski [53], Mueller [54], Benicewicz [55], Takahara [56], Hawker [57] and coworkers have paved the road for progress in SI-CRP methods. SI-CRPs (ATRP, SI-RAFT and SI-NMP) have been successfully employed for the generation of plethora of polymer grafted nanoparticles (PGNPs) because of its tolerance towards various functional groups [48]. 

Table 1 summarizes the advantages and disadvantages of the four approaches outlined in the synthesis of PGNPs. Among the various approaches, grafting from approach is widely employed in the polymer functionalization of nanoparticles because of its ability to synthesize well-defined polymer architectures of desired composition and molecular weight, and a shell of controlled thickness on the nanoparticle surface. Given the enormous data available on grafting from technique, this article will predominantly cover this approach. Examples of other approaches in the polymer functionalization of ceramic oxide NPs are also covered.

## 2. Grafting from Approach

**Figure 3:**
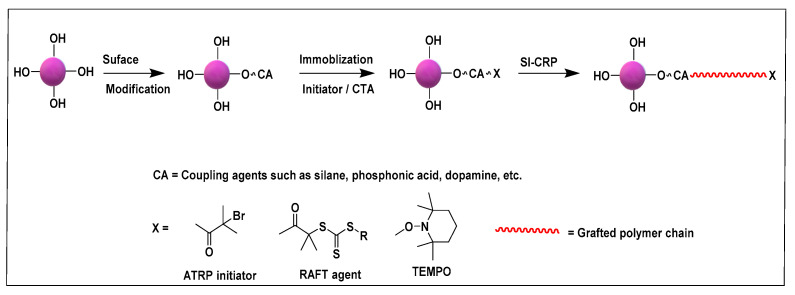
Scheme for surface initiated controlled radical polymerization methods.

Grafting from approach may entail the use of anionic or cationic or free radical polymerization in the functionalization of NPs. SI-anionic and cationic polymerizations are excellent routes in providing polymer grafted nanoparticles (PGNPs) with predetermined molecular weights of narrow dispersity [60,61,62,63]. However, the complexity of the experimental techniques limits their broad use [64,65,66,67]. Alternatively, initiator immobilized NPs have been subjected to free radical polymerization to yield graft NPs [68,69]. Conventional free radical polymerization suffers from poor control of molecular weights, chain-end functionality, and polydispersity [31]. Therefore, surface initiated controlled radical polymerization techniques such as atom transfer radical polymerization (ATRP), reversible addition−fragmentation chain-transfer polymerization (RAFT) and nitroxide mediated polymerization (NMP) have been pursued for the synthesis of well-defined PGNPs. Controlled radical polymerization (CRP) technique involves reversible activation–deactivation equilibrium between active chain propagating species and dormant species which lower the rate of chain propagation than that of conventional free radical polymerization. Thus, CRP polymerization offers a route to synthesize PGNPs with well-defined molecular weights and low dispersity. Figure 3 gives a general scheme for the various SI-CRPs methods. Typically, the synthesis of PGNPs is based on the surface modification of NPs, then anchoring/immobilization of initiator/chain transfer agent attachment on the surface-modified NPs and finally polymerization using the surface initiator attached to NPs to obtain PGNPs. Surface modification of NPs is often accomplished with coupling agents such as silane, phosphonic acids, dopamine, etc. More details about the surface modification of NPs with reagents such as silane agent, phosphonic acid, and dopamine can be found in our recent review article [14]. The second step is introducing initiator functionality on the surface agent modified NPs. Alternatively, initiator functionality and coupling agent are pre-reacted to form initiator functionalized coupling agent which is then subsequently reacted with NPs [70]. The merits and demerits of various surface initiated controlled radical polymerization techniques have been presented in Table 2.

## 3. Atom Transfer Radical Polymerization (ATRP)

Atom transfer radical polymerization (ATRP) is one of the most versatile polymerization techniques adopted towards the synthesis of PGNPs because the technique can be used under broad experimental conditions and can be adapted to synthesis of polymers with a wide range of functional groups [74,75]. The polymerization of activated vinyl monomer by ATRP process generally requires alkyl halide initiator and a transition metal complex as catalyst (e.g., CuBr/ligand). ATRP involves reversible activation—deactivation equilibrium between a metal-ligand complex and halide end-capped chain to form radical species which propagates the polymerization. Mechanistic details of ATRP can be found in the literature [71,74,76,77,78]. 

Several modifications to ATRP have been studied such as, activator regenerated by electron transfer ATRP (ARGET ATRP), reverse ATRP, UV Light mediated ATRP, and electrochemical mediated ATRP, etc. In ARGET ATRP a reducing agent viz., 2-ethylhexanoate or ascorbic acid or glucose is employed to regenerate the active transition metal complex via reduction of the higher oxidation state transition metal complex [79]. On the other hand, “reverse” ATRP consists of the addition of transition metal complexes in the higher oxidation state and the generation of the lower oxidation state activator by reaction with a conventional free radical initiator [76,80,81]. Initially, alkyl halide initiators are immobilized onto the NP surface. Using CuBr/ligand system, the polymerization proceeds like the classical ATRP polymerization in bulk or solution and monomers are polymerized on the surface of the NPs in a controlled manner. 

### 3.1. SI-ATRP Polymerization to Prepare Polymer-Grafted SiO_2_ Nanoparticles

**Figure 4:**
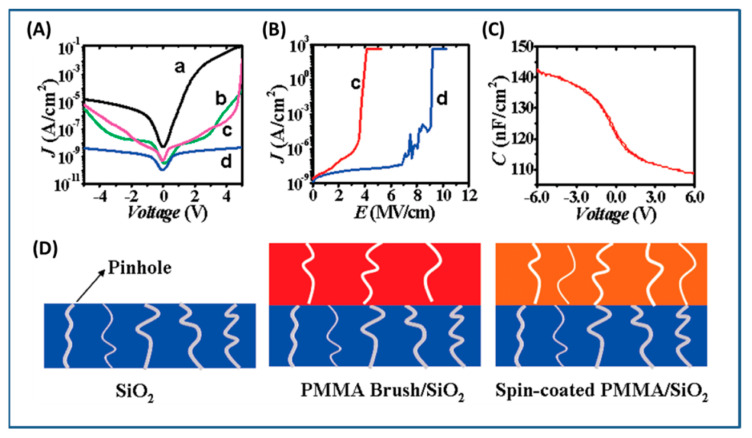
(**A**) Leakage characteristics and (**B**) breakdown electric field characteristics for different dielectrics measured with the structure of Au/dielectric/Si capacitor. Curve a, SiO_2_ (9 nm); curve b, spin-coated PMMA (10 nm)/SiO_2_ (9 nm); curve c, surface-grafting PMMA (20 nm); curve d, surface-grafting PMMA (10 nm)/SiO_2_ (9 nm). (**C**) Capacitance-voltage characteristics for PMMA/SiO_2_ dielectrics. *C*-*V* curves were measured at an ac signal frequency of 1 MHz. (**D**) Schematic diagram of distribution of pinhole defect in the dielectrics, indicating the reason why PMMA brush/SiO_2_ bilayer dielectrics show the lowest the leakage compared bare SiO_2_ and spin coated PMMA/SiO_2_ dielectrics. Reproduced with permission from Ref. [101].

ATRP reactions have been extensively used to grow polymer/block copolymer brushes from the surface of silica with controlled graft densities [82,83,84]. For example, polymer/copolymer brushes of PMMA [83,85], polystyrene (PS) [86,87], poly(glycidyl methacrylate) (PGMA) [88,89], poly(2-hydroxyethyl methacrylate) (PHEMA [90], poly(4-vinylpyridine) (PVP) [91], poly(N-isopropylacrylamide) (PNiPAAm) [92], poly(sodium 4-styrene sulfonate) (PSS) [93], poly((ethylene glycol)methyl ether methacrylate) (POEGMA) [94], poly(2-(dimethylamino)ethyl methacrylate) (PDMAEMA) [95,96], etc. have been successfully grafted on SiO_2_ surface via SI-ATRP. Pinto et al. [97] employed SI-ATRP for grafting of PMMA brushes thinner than 50 nm on SiO_2_ substrate for tunnel emitter transistor application at operating voltage below 5 V (which is an important requirement for industrial adoption). Hwang et al. [98] employed SI-ATRP for grafting PS brushes on silica surface with controlled molecular weight (24,600–135,000 g/mol) as well as grafting density (0.34–0.54 chains/nm^2^). The performance of pentacene-based thin-film transistor fabricated from PS-grafted SiO_2_ as a gate dielectric was evaluated as a function of polymer brush thickness viz. 12.4, 47.5 and 113.1 nm. The device fabricated from 47 nm thickness of PS brush exhibited highest mobility (µ_FET_ = 0.099 cm^2^/V·s) indicating that optimum molecular weight polymer brushes need to be grown from the surface of dielectric for achieving best performance. The OTFTs with the PS-grafted SiO_2_ layer showed 2 times higher mobility (µ_FET_ = 0.099 cm^2^/V·s) than that of bare SiO_2_ layer (µ_FET_ = 0.05 cm^2^/V·s). The electrode/active layer interface showed enhanced mobility which could be attributed to grafted PS influencing the morphology of pentacene by enhancing the crystalline structure [98]. Li and coworkers synthesized PMMA-*g*-SiO_2_ NPs with ~10 nm PMMA brush onto the SiO_2_ layer (~9 nm) via SI-ATRP. PMMA brush/SiO_2_ bilayer dielectrics showed the lowest leakage compared to bare SiO_2_ and spin coated PMMA/SiO_2_ dielectrics which could be attributed to improved interfacial morphology, a smaller number of pinholes at the interface due to the close packing of polymer brush (Figure 4). The surface-grafted PMMA brush (10 nm)/SiO_2_ (9nm) on silicon wafer exhibited lower leakage and higher breakdown strength than that of surface-grafted PMMA brush (20 nm) on silicon wafer (free of 9 nm SiO_2_ layer) (Figure 4A,B). The authors attributed the enhancement in the breakdown strength of PMMA brush (10 nm)/SiO_2_ (9 nm) on silicon wafer over PMMA brush (20 nm) grafted on silicon wafer (free of 9 nm SiO_2_ layer) due to the presence of bilayer and improved interaction between polymer brush and SiO_2_ layer [99,100]. The PMMA-*g*-SiO_2_ nanodielectric exhibited good operational stability, and good compatibility with organic semiconductors, which enabled OFETs to work at high performance and low voltage [101]. 

Similar observations were also made by Li and coworkers by operating copper phthalocyanine (CuPc) transistors at an operational voltages of 2.0 V using surface-grafted ~10 nm PMMA brush on silica [70]. Additionally, it was noted that the thickness of the polymer brush on silica could be modulated based on the activity of the catalyst, the reactant concentration and reaction time. The PMMA brushes on silica showed high-quality dielectric property, including excellent insulating characteristics, large capacitance, and low charge-trapping density. Field-effect transistors with PMMA brush as the dielectric layer demonstrate excellent charge transport. Table 3 summarizes dielectric and electronic properties of transistors fabricated from surface-grafted polymer brushes.

### 3.2. SI-ATRP Polymerization to Prepare Polymer-Grafted TiO_2_ Nanoparticles

ATRP has also been widely used to grow PMMA [102,103,104,105,106], PS [107,108,109,110], poly(styrene sulfonic acid) (PSSA) [111,112], poly(oxyethylene methacrylate) (POEM) [113,114], PNIPAAm, [115,116], PHEMA [117] on the surface of TiO_2_. For example, Krysiak et al.; [118] performed the SI-ATRP grafting of poly(di (ethylene glycol) methyl ether methacrylate) on the surface of TiO_2_ (rutile) so as to yield polymer brushes with thickness of 10–15 nm (as measured by TEM) and molecular weight, Mn of ~60,000 g/mol. Similarly, Park et al. [114] utilized ATRP for the synthesis of TiO_2_ nanoparticles grafted with POEM and PSSA. In the first step, the -OH groups on the surface of TiO_2_ nanoparticles were converted to -Cl groups by the reaction of TiO_2_ with 2-chloropropionyl chloride (CPC) (ATRP initiator) which was used to initiate POEM and PSSA grafting on the surface of the TiO_2_ nanoparticles. The modified TiO_2_ nanoparticles showed better dispersion in alcohol than unmodified nanoparticles. X-ray diffraction (XRD) studies of polymer-grafted-TiO_2_ nanoparticles revealed that there was no significant change in the crystalline structure of the TiO_2_ nanoparticles. There are number of reports on utilization of SI-ATRP for grafting of polymer on TiO_2_ nanoparticles, however no significant studies have been reported on the dielectric properties of SI-ATRP polymer grafted TIO2 nanoparticles filled polymer nanocomposites. 

### 3.3. SI-ATRP Polymerization to Prepare Polymer-Grafted BaTiO_3_ Nanoparticles

**Figure 5:**
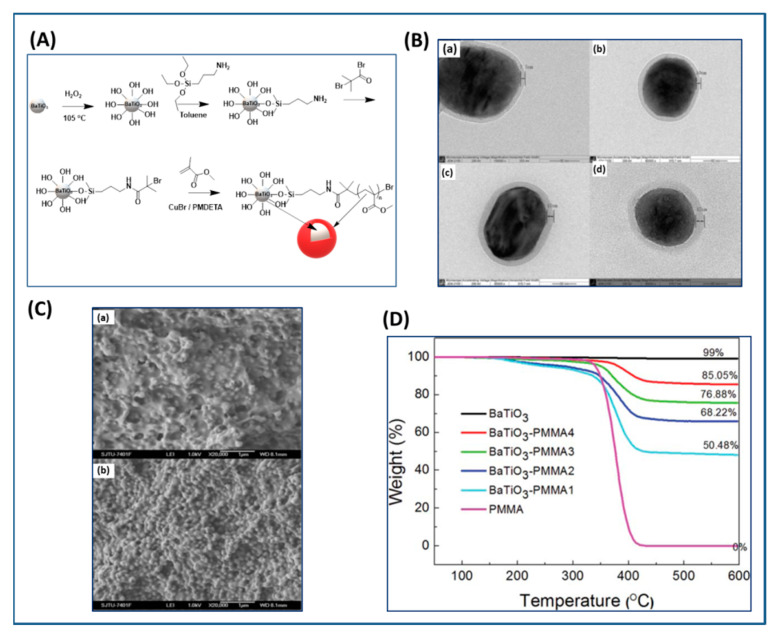
(**A**) Schematic diagram illustrating ATRP approach of growing PMMA from BaTiO_3_ (**B**) TEM images of PMMA-BaTiO_3_ (**a**), PMMA3-BaTiO_3_ (**b**) PMMA2-BaTiO_3_ (**c**), PMMA1-BaTiO_3_ (**d**). (**C**) SEM of the cross-sectional images of composite films: PMMA1-BaTiO_3_ (**a**) and PMMA2-BaTiO_3_ (**b**). (**D**)TGA curves for the pure PMMA and PMMA-BaTiO_3_. Reproduced with permission from Ref. [119].

The initial reporting about the use of SI-ATRP approach to graft polymer on BaTiO_3_ nanoparticles was based on performing hydroxylation, sialylation, grafting of the anchoring group, followed by chain growth polymerization [119]. Table 4 summarizes the conditions used to synthesize various polymer-grafted BaTiO_3_ nanoparticles. Figure 5 presents the scheme for synthesis of PMMA-grafted BaTiO_3_ nanoparticles. This study showed that the thickness of the PMMA shell could be varied by changing the feed ratio of BaTiO_3_ (76% to 0%) to MMA resulting in grafted nanoparticles with dielectric constant ranging from 14.6 to 3.49 (pure PMMA). The PMMA-grafted BaTiO_3_ nanoparticles showed dielectric loss below 0.04, which was slightly lower than that of PMMA. 

**Figure 6:**
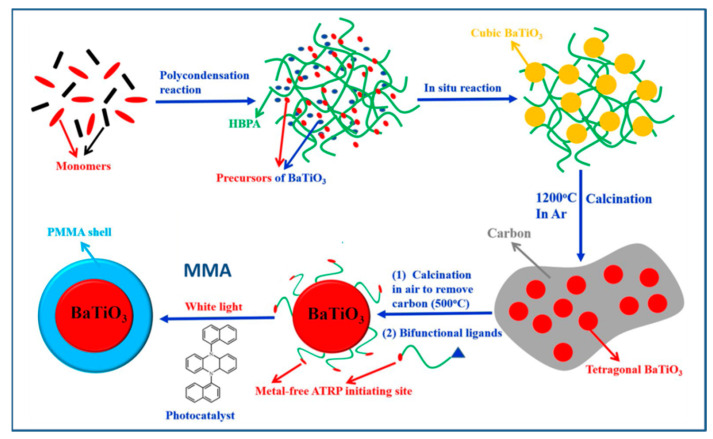
Scheme for the preparation of core/shell ferroelectric BaTiO_3_/PMMA hybrid nanoparticles by metal-free ATRP process driven by visible light based on novel hyperbranched aromatic polyamides (HBPA) as functional matrix. Reproduced with permission from Ref. [120].

Likewise, You et al. [120] demonstrated an approach to tune the dimension of BaTiO_3_ nanoparticles and vary the polymer shell thickness using ATRP method in the absence of metal catalyst. Initially, the BaTiO_3_ nanoparticles were formed by polycondensation of precursors (barium hydroxide (Ba(OH)_2_) and titanium(IV) tetraisopropoxide (Ti(OiPr)_4_) and HBPA) followed by calcination. (Figure 6) The NPs were then modified by bi-functional ligands (12-hydroxydodecanoic acid and 2-bromophenylacetyl bromide) followed by MMA polymerization using white light and photocatalyst. Using this approach, the authors demonstrated that the dimensions of BaTiO_3_ nanoparticles could be adjusted based on the molar ratio of HBPA and precursors, while the thickness of polymeric shell could be adjusted based upon the duration of white LED irradiation. The dielectric properties of core/shell BaTiO_3_/PMMA hybrid nanoparticles were found to depend upon the dimension of BaTiO_3_ core and the molecular weight of PMMA shell. For example, the dielectric constant of core/shell BaTiO_3_/PMMA hybrid nanoparticles with larger core size (core size: ~39 nm, ε = 22.23 ± 1.09, shell thickness: 6 nm) was found to be higher than that of smaller core size sample (core: ~17 nm, ε = 17.06 ± 0.58, shell thickness: 6 nm). This is due to the increased contribution of BaTiO_3_ to the overall dielectric constant with increase in the core size of BaTiO_3_ and changes in the crystallinity from cubic (paramagnetic) to tetragonal (ferromagnetic). Similarly, the dielectric constant of core/shell BaTiO_3_/PMMA hybrid nanoparticles with varying molecular weight of PMMA shell were studied and it showed an inverse relationship to the thickness of the PMMA shell. For example, the dielectric constant of core/shell BaTiO_3_/PMMA hybrid nanoparticles with smaller shell thickness (shell thickness: 6 nm core size ~39 nm, ε = 22.23 ± 1.09) was found to be higher than that of larger shell thickness (shell thickness: 8 nm core size ~39 nm, ε~13). This is because larger shell thickness corresponds to the higher proportion of PMMA contribution to the overall dielectric constant of core-shell nanoparticles, especially given that PMMA has lower dielectric constant than that of core BaTiO_3_.

**Figure 7:**
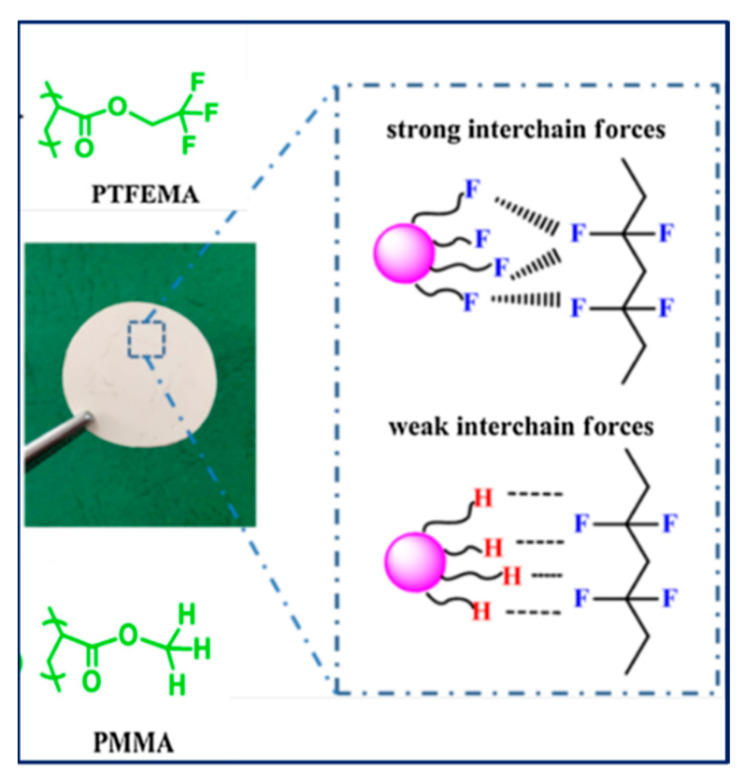
Fluorine-fluorine and hydrogen-fluorine interactions in the nanocomposites of PVDF and BaTiO_3_ grafted with PTFEMA and PMMA, respectively. Reproduced with permission from Ref [121].

Apart from BaTiO_3_ core size, polymer shell thickness, also the composition of polymer shell can influence the dielectric properties of nanocomposites. In this regard, Zhang et al. [121] studied core-shell structured PMMA@BaTiO_3_ (brush thickness, 7–12 nm) and PTFEMA@BaTiO_3_ (brush thickness, ~5 nm) nanoparticles that were synthesized by reacting (3-aminopropyl) trimethoxysilane (APTMS) and α-bromoisobutyrylbromide (BIBB) with BaTiO_3_ nanoparticles followed by reaction with methyl methacrylate (MMA) or 1,1,1-trifluoroethyl methacrylate (TEFMA). At 1:1 weight feed ratio, (BaTiO_3_ and MMA or TFEMA), the polymer brush thickness for PMMA@BaTiO_3_ and PTFEMA@BaTiO_3_ was found to be 7 nm and 4.5 nm, respectively with grafting density of 5.5% and 1.5%, respectively. MMA formed larger shell due to its enhanced reactivity than TFEMA. The study of the dielectric properties of PMMA@BaTiO_3_ and PTFEMA@BaTiO_3_ exhibited significant improvement in dispersity of polymer-grafted BaTiO_3_ nanoparticles in polyvinylidene fluoride (PVDF) matrix leading to decreased dielectric loss. Furthermore, PMMA@BaTiO_3_/PVDF and PTFEMA@BaTiO_3_/PVDF nanocomposites exhibited attenuation of dielectric constant of 16.6% and 5.5% at grafted density of 5.5% and 1.5%, respectively compared to controls. A comparison of the performance of PTFEMA@BaTiO_3_ nanoparticles in PVDF matrix showed 90% decrease in dielectric loss as compared to BaTiO_3_/PVDF while PMMA@BaTiO_3_ nanoparticles/PVDF nanocomposites showed 80% decrease in dielectric loss as compared to BaTiO_3_/PVDF. This could be attributed to the stronger interaction between PFTEMA with PVDF matrix resulting in an enhancement in the interfacial polarization and stabilization of electric field (Figure 7).

**Figure 8:**
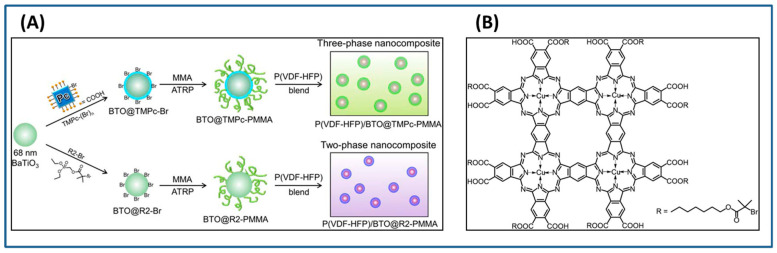
(**A**) Schematic illustration of the preparation of three- and two-phase P(VDF-HFP)/BaTiO_3_ nanocomposites, respectively; (**B**) Chemical structure of the TMPc-Br ATRP initiator. Reproduced with permission from Ref. [124].

Alternatively, PMMA can be grafted on BaTiO_3_ nanoparticles by coating of a highly polarizable tetrameric metallophthalocyanine (TMPc) as ATRP initiator on the surface of BaTiO_3_ nanoparticles instead of conventional ATRP initiator followed by polymerization of MMA (Figure 8). As control, R2-PMMA@BaTiO_3_ nanoparticles without TMPc interfacial layer were synthesized via phosphonate coupling of (R2-Br) followed by ATRP polymerization of MMA. Due to the high polarizability of the TMPc interfacial layer and the high dielectric constant of TMPc [122,123], poly(vinylidene fluoride-co- hexafluoropropylene) (PVDF-HFP)/PMMA-TMPc@BaTiO_3_ films exhibited higher dielectric constant (26% higher than nanocomposite without TMPc), and improved higher energy density (20% higher than neat (PVDF-HFP)) at nanofiller filling ratios of 4.69 vol% [124]. 

**Figure 9:**
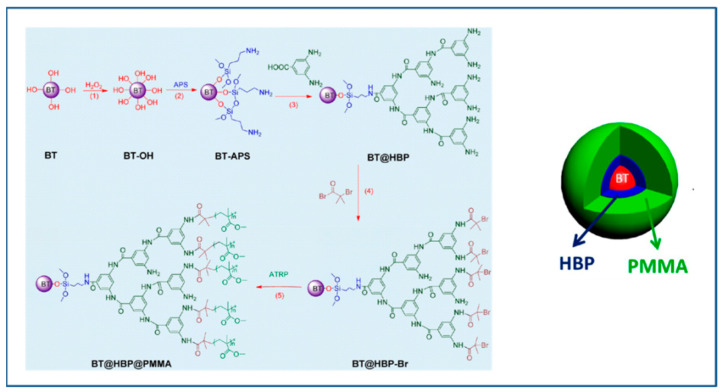
Schematic illustrating the preparation of PMMA@HBP@BT. Reproduced with permission from Ref. [125].

Xie et al. [125] synthesized a core@double-shell structured PMMA@HBP@BT nanocomposite via a two-step process as depicted in Figure 9. In the first step, the hyperbranched aromatic polyamide was grafted on the surface of BaTiO_3_ nanoparticles, and in the second step, the hyperbranched amine was used for grafting of PMMA shell via SI-ATRP. The thickness of the second shell was controlled by adjusting the ratio of MMA and macro initiator, BT@HBP-Br. The SEM morphology of PMMA@HBP@BT revealed improved adhesion between BT nanoparticles and polymers (HPB and PMMA, covalently attached) as compared to BT@HBP/PMMA nanocomposite. The PMMA@HBP@BT/PMMA nanocomposite exhibited high dielectric constant (39.3, 10 times higher than that of PMMA) as well as low dielectric loss (0.0276). The nanocomposite of BT@HBP in PMMA matrix (56.7% loading) resulted in high dielectric constant of 113 while loss was increased to 0.485 (16.6 times higher than that of PMMA@HBP@BT). Thus, double core-shell structured PMMA@HBP@BT provides another approach for preparing nanocomposites with higher dielectric constant and low dielectric loss. 

**Figure 10:**
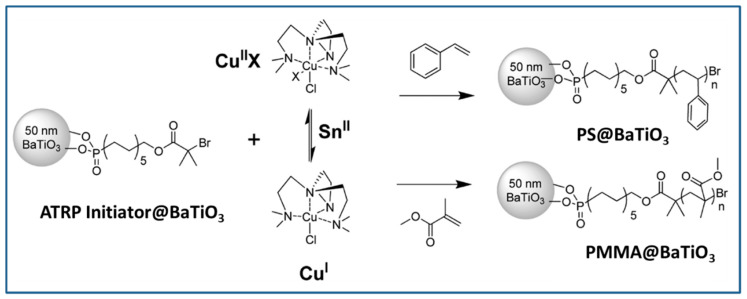
Surface-initiated polymerization of styrene or methyl methacrylate via ARGET ATRP. Reproduced with permission from Ref. [27].

The attachment of phosphonic acid-based ATRP initiator on BaTiO_3_ nanoparticles followed by growth of PMMA on BaTiO_3_ nanoparticles via activated regenerated by electron transfer (AGRET) ATRP approach (Figure 10) was reported to compare and contrast the dielectric performance of single- and multi-component nanocomposites [27]. A comparison of PMMA@BaTiO_3_ one component nanocomposite and phosphonic acid-modified BaTiO_3_ mixed PMMA two component nanocomposite, at same loading of 16 vol%, showed that the two-component nanocomposite has energy density of ~1.9 J/cm^3^ at 256 V/μm while one component nanocomposite has energy density of ~2 J/cm^3^ at a 25% lower field strength (220 V/μm) which implies a 2-fold enhancement in energy density due to the covalent attachment of PMMA to BaTiO_3_ nanoparticles.

Zhang et al. [126] synthesized core-shell structured poly(1H,1H,2H,2H-perfluorooctyl methacrylate) (PPFOMA )@BaTiO_3_ nanoparticles via SI-ATRP and was used for the formulation of single component nanocomposite. One of the distinct advantages of single component nanocomposite is the ability to load high % of ceramic nanofiller with minimal effect on dispersibility. The dielectric properties of single component nanocomposites (various core-shell nanoparticles were formed by changing the feed ratio of PFOMA and BaTiO_3_) were evaluated over a broad frequency from 40 Hz to 30 MHz at room temperature. The results revealed that the dielectric constant (k) increased and dielectric loss reduced significantly with the addition of BaTiO_3_. The k of the composite was up to 7.4 at 100 kHz at room temperature when the BaTiO_3_ loading was up to 70 wt% which is almost three times greater that of pure PPFOMA (k = 2.6). However, the dielectric loss (0.01) of PPFOMA@BaTiO_3_ composite of one component polymer nanocomposite for 70 wt% loading was much lower than that of the pure PPFOMA (0.04). It is interesting to highlight that the nanocomposite despite high loading of nanofiller exhibited low loss even less than that of pure polymer at 70% nanoparticle loading.

The structure-property relationship study of polymer-grafted BaTiO_3_ nanoparticles (synthesized by ATRP technique) filled polymer nanocomposites [27,119,120,121,124,125,126] clearly indicates that several factors influence the dielectric performance of the nanocomposite including the thickness of the core and the shell of the core-shell nanoparticles and the type of polymer-grafted on the nanoparticles, interfacial separation between core NPs and polymer shell, the composition of nanocomposite (single or multicomponent type of nanocomposite), the type of interfacial layer and double shell coverage of nanoparticles.

### 3.4. SI-ATRP Polymerization to Prepare Polymer-Grafted Al_2_O_3_ Nanoparticles

Sanchez et al., [128] reported the modification of aluminum oxide nanoparticles by poly(lauryl methacrylate) (PLMA) using surface-initiated ATRP (SI-ATRP) technique. The molecular weight of grafted polymer ranged between 23,000 and 83,000 g/mol. PLMA-grafted nanoparticles filled LDPE matrix resulted in lower dielectric loss-tangent (~0.0008 to ~0.0003 with 1 wt% at 100 Hz) compared to LDPE filled with bare Al_2_O_3._ This may be a result of the enhanced adhesion between LDPE and the lauryl chains of the grafted polymer on the nanoparticles. Table 5 summarizes of dielectric properties of polymer nanocomposites fabricated from polymer brushes-grafted ceramic nanoparticles obtained using different grafting techniques.

## 4. Reversible Addition−Fragmentation Chain-Transfer Polymerization (RAFT)

Living free-radical polymerization by reversible addition−fragmentation chain transfer (RAFT), is one of the most versatile and powerful technique for controlled radical polymerization which was invented in 1998 by Moad and co-workers [143] RAFT polymerization involves a degenerative chain transfer method to control polymerization [144] unlike ATRP and NMP which has a persistent radical in the system [76,145,146,147]. The control in RAFT polymerization is derived from the chain transfer agent (CTA) and the details about RAFT mechanism can be found in the following references [144,148,149,150]. In comparison to other CRPs, RAFT polymerization has number of advantages such as being adaptable to almost all free radical polymerizable monomers, the ability to synthesize multi-block copolymers with a high degree of fidelity, the ability to work in the presence of oxygen, no need of inorganic catalysts and mild polymerization conditions, similar to that of conventional free radical polymerization [151,152,153,154,155,156]. SI-RAFT has been widely used for the preparation of polymer-grafted nanoparticles by attaching CTA functionality to the surface of nanoparticles. In SI-RAFT polymerization, the attachment of the CTA moiety to the NP surface could be done via “Z” group or “R” group. If the NP is attached to the “Z” group of the CTA then growing polymer chains will detach propagate, and then reattach to the NP surface, just like a “graft to” approach [157,158]. Thus, “Z” group attachment of CTA lead to decreased graft density because of the bulky nature of the polymer chains being grafted to nanoparticles using graft to approach. However, if the NP is attached to the “R” group of the CTA then the monomer gets sequentially added to the propagating polymer radicals present on the NP surface. This approach is the preferred pathway to synthesize core-shell NPs using SI-RAFT. Table 6 summarizes some examples of the anchored CTA structures and polymerization conditions used to synthesize various polymer grafted nanoparticles via SI-RAFT.

### 4.1. SI-RAFT Polymerization to Prepare Polymer-Grafted SiO_2_ Nanoparticles

A variety of polymers such as PMMA, PS, PNiPAAm, PAA, PHEMA, P4VP, polyisoprene have been grown from the surface of silica nanoparticles through ”grafting from” approach via SI-RAFT polymerization [48,159,160,161,162,163,164,165]. For example, the amino-functionalized SiO_2_ (SiO_2_–NH_2_) nanoparticles served as the precursor for RAFT polymerization and were synthesized by reacting amino propyl triethoxysilane (APTES) with the bare SiO_2_ nanoparticles. Subsequently, the RAFT-CTA viz, 4-cyano-4-(dodecylsulfanylthiocarbonyl) sulfanyl pentanoic acid (CDP) agent was immobilized on the surface of SiO_2_–NH_2_ nanoparticles by amide forming reaction. The CDP immobilized SiO_2_ nanoparticles were then used in the surface-initiated RAFT polymerization of HEMA with AIBN as the free radical initiator, to form PHEMA-*g*-SiO_2_ nanoparticles [164]. 

There are several other examples of immobilization of RAFT-CTA on the surface of SiO_2_ nanoparticles. For example, dopamine is reacted with silanized nanoparticles followed by dicyclohexyl carbodiimide (DCC) coupling, [160] or silanization of nanoparticles with modified RAFT-CTA agent where the RAFT-CTA agent was precoupled with silane agent [166,167,168], or silanization of nanoparticles with chloro functionality so as to eventually react with sodium/potassium ethyl xanthate to form xanthate [162,163]. 

**Figure 11:**
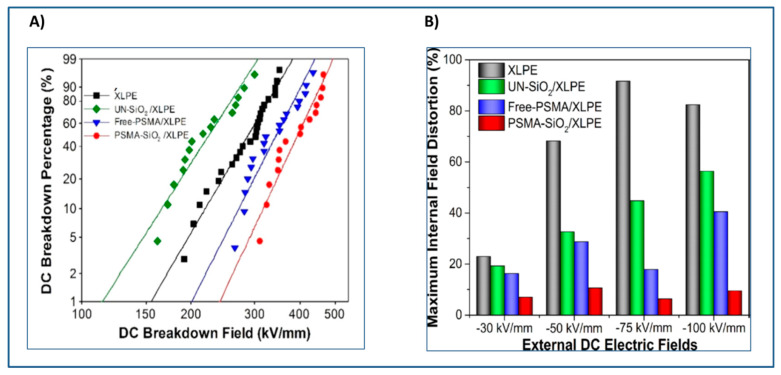
(**A**) Weibull distribution of DC breakdown strength, (**B**) Maximum internal field distortion under external DC electric fields. Reproduced with permission from Ref. [177].

Literature presents several examples of the use of unimodal polymer grafted SiO_2_ nanoparticles to enhance the breakdown strength of polymer grafted SiO_2_ filled nanocomposites [177,178,180]. For example, SI-RAFT technique has been used in the synthesis of poly(stearyl methacrylate) (PSMA) (Mn = 45 kg/mol and the graft density = 0.04 chain/nm^2^) grafted SiO_2_ (10–15 nm diameter) nanoparticles [177]. The dielectric performance of PSMA-grafted SiO_2_ nanoparticles/XLPE was compared with XLPE, pure PSMA/XLPE and unmodified SiO_2_/XLPE. Among the systems evaluated, the unmodified SiO_2_ nanoparticles filled/XLPE exhibited lowest dielectric breakdown strength while PSMA grafted SiO_2_ nanoparticles dispersed in XLPE showed the highest dielectric breakdown strength. (Figure 11A) The internal field distortion of PSMA grafted SiO_2_ nanoparticles in XLPE was found to be the least (less than 10.6%) among the nanocomposites (Figure 11B) studied over a wide range of DC fields from −30 kV/mm to −100 kV/mm indicating tremendous potential for improving HVDC power cable insulation. The long alkyl chain of PSMA present on nanoparticles appears to have enhanced the interaction of nanoparticles with XLPE matrix hence the improved breakdown strength of nanocomposite [177].

**Figure 12:**
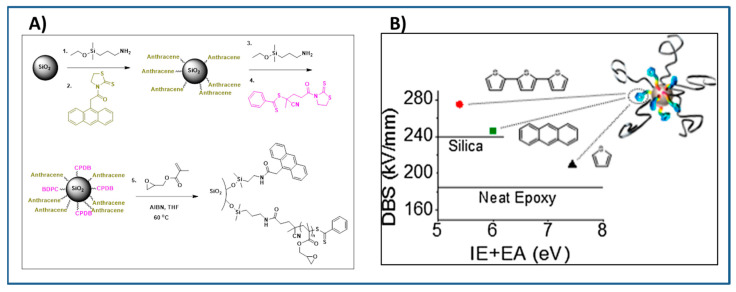
(**A**) Synthesis of bimodal anthracene-PGMA silica nanoparticles (**B**) calculated summation of ionization energy and electron affinity for electroactive ligands V·s. experimentally determined dielectric breakdown strength of several bimodal composites. Reproduced with permission from Ref. [187].

The use of bimodal polymer grafted nanoparticles in polymer nanocomposite offers an attractive alternative approach for achieving improved breakdown strength and better nanoparticle dispersion in polymer nanocomposites. The synthesis of bimodal polymer grafted nanoparticles was explored by Benicewicz, Schadler and coworkers [181,182,183,184]. Bimodal polymer grafted SiO_2_ nanoparticles were synthesized by sequential attachment of electroactive conjugated surface ligands followed by surface-initiated RAFT polymerization of GMA (Figure 12) to form PGMA. The electroactive functionality (anthracene, thiophene, and terthiophene) was also grafted on the nanoparticles. Grafting of conjugated molecules (anthracene, thiophene and terthiophene) to the nanoparticle surface offers an approach to promote electron trapping at isolated regions of the composite while restricting the formation of conductive pathway [185], while the grafted PGMA chains promoted improved dispersion of the multifunctional SiO_2_ nanoparticles in epoxy resin. Bimodal terthiophene-PGMA functionalized SiO_2_ nanoparticles filled composites showed the highest enhancement in dielectric breakdown strength followed by bimodal anthracene-PGMA functionalized nanoparticles filled epoxy sample and the least was for thiophene-PGMA functionalized nanoparticles filled epoxy sample. The role of substituted aromatics grafted on nanoparticles in improving the dielectric breakdown strength of nanocomposite was explained on the basis of the Hammett relationship [186]. 

Similarly, bimodal anthracene-PSMA grafted SiO_2_ nanoparticles were dispersed in polypropylene. The dispersion of bimodal modified SiO_2_ nanoparticles in polypropylene resulted in the enhancement of dielectric permittivity by 20% and an improvement in the dielectric breakdown strength under both AC and DC test conditions by about 15% compared to neat polypropylene [178]. As noted earlier, the long alkyl chain of PSMA on grafted nanoparticles appears to have improved the compatibility of nanoparticles with XLPE matrix hence the improved dielectric properties of nanocomposites.

Alternatively, the bimodal functionalized nanoparticles can be synthesized with long brushes of PS chains and short P2VP chains using SI-RAFT technique. The combined effect of interaction of PS brushes of the grafted nanoparticles with the matrix and the reduction in silica core-core NPs interaction because of the dense short grafts of P2VP present in the grafted nanoparticles, contributed to the improved dispersion of nanoparticles in PS matrix. Unlike the earlier papers on bimodal grafted nanoparticles which dealt with dielectric properties of nanocomposites, the emphasis of Kumar et al. publication [188] was on the dispersion of nanoparticles in polymer matrix and the impact of microstructure on the mechanical properties of nanocomposites. 

### 4.2. SI-RAFT Polymerization to Prepare Polymer Grafted TiO_2_ Nanoparticles

**Figure 13:**
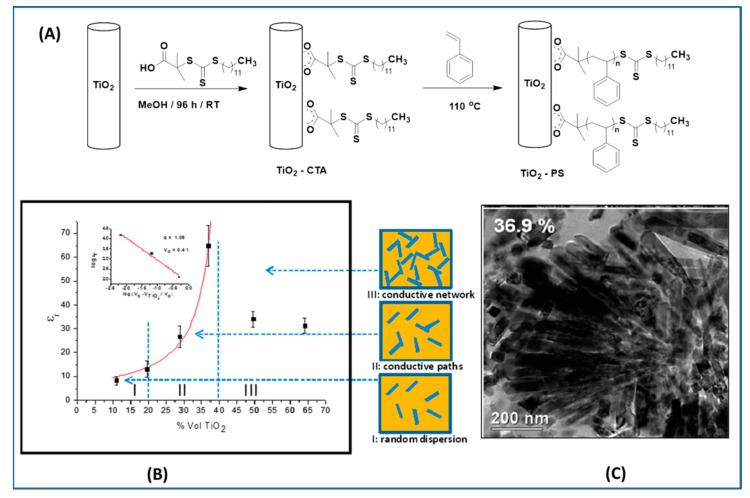
(**A**) Schematic diagram illustrating the process of RAFT polymerization on the surface of rutile nanoparticles; (**B**) Variation of the dielectric constant of the PS@TiO_2_/PS composite V·s. the volume fraction of TiO_2_ at 10^4^ Hz; (**C**) TEM micrograph of the TiO_2_–PS composites (at 36.9% TiO_2_ content). Reproduced with permission from Ref. [138].

SI-RAFT has been employed for growing polymers such as PMMA [189,190], PS [138], polyacrylic acid (PAA) [191,192], poly(n-vinylpyrrolidone) [193], poly(chloromethyl styrene) [194], poly(2-hydroxyethyl acrylate) [195], PMMA-*b*-PS, [179,196], etc. on the surface TiO_2_. PS (Mn = 4800 g/mol) was grown from rutile TiO_2_ nanoparticles via SI-RAFT polymerization and dispersed in PS matrix at various concentrations to investigate the dielectric properties of nanocomposites. The PS chains attached to the surfaces of TiO_2_ (PS@TiO_2_) nanoparticles maintained a “brush-like” structure and resulted in chestnut-burr (Figure 13C) self-assembled NP aggregates. With increase in the amount of PS@TiO_2_-, the composite showed a higher dielectric constant (~65) which could be attributed to the self-assembled chestnut-burr aggregates of the nanoparticles where a number of rutile crystals shared lateral faces and formed capacitive microstructures. The crystals in these aggregates are separated by a polymer thin layer and allow a high percolation threshold, 41% *v/v* of filler amount, before the formation of a continuous network responsible for the sudden change of the dielectric characteristics, (from random orientation to conductive pathways to conductive network) as depicted in Figure 13. Despite the high content of inorganic filler, the dissipation factor remained low, even approaching the lower frequencies. 

### 4.3. SI-RAFT Polymerization to Prepare Polymer Grafted BaTiO_3_ Nanoparticles

**Figure 14:**
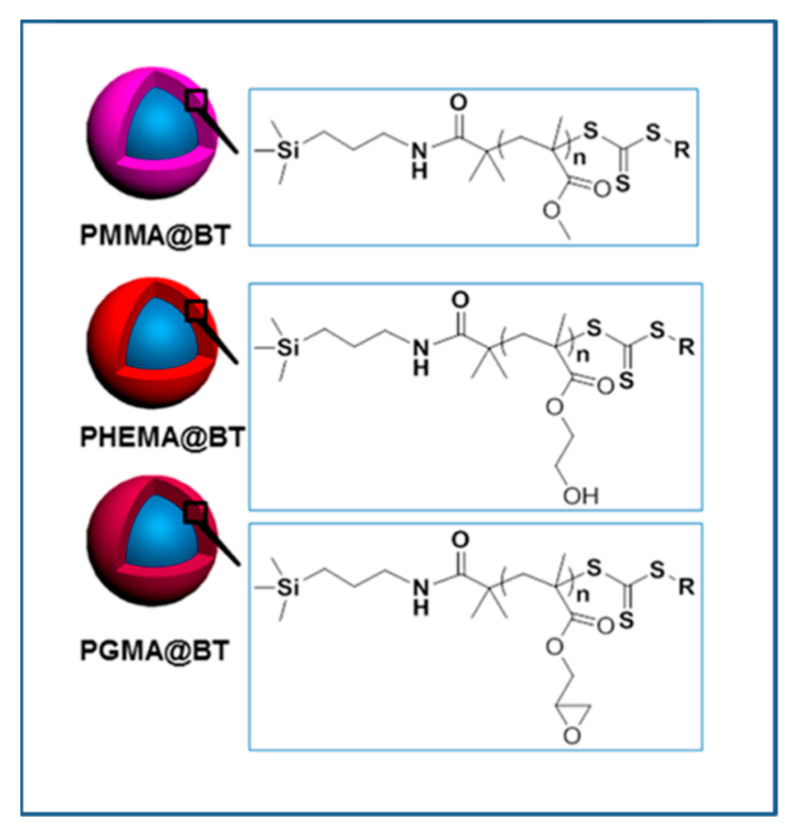
BaTiO_3_ core-shell nanoparticles by surface-initiated RAFT polymerization. Reproduced with permission from Ref. [197].

Ming Zhu et al. [197] synthesized core-shell structured polymer@BaTiO_3_ nanoparticles of varying polymer composition (PMMA@BT, PGMA@BT, and PHEMA@BT) and constant shell thickness (Figure 14) using SI-RAFT technique. The synthesized nanoparticles were used to study the compositional effect of the polymeric shells of PGNPs on the dielectric properties of the nanocomposites i.e., breakdown strength, leakage currents, energy storage capability, and energy storage efficiency of the nanocomposites. The differences in the dielectric properties of the various core-shell NPs (with PHEMA, PGMA and PMMA shell) were attributed to the differences in the dipole moment of pendant groups in the shell. The hydroxyethyl pendant group in PHEMA was responsible for the larger dipole moment and higher moisture absorption, resulting in the higher dielectric constant and higher loss as compared to PGMA and PMMA. Among the systems studied, PHEMA@BT/PVDF nanocomposite exhibited highest storage energy density due to the high dielectric constant of PHEMA@BT while the PGMA@BT/PVDF nanocomposite exhibited highest discharge density due to the high breakdown strength and low dielectric loss of PGMA@BT (20% loading), while PMMA@BT/PVDF (20% loading) nanocomposite exhibited highest energy storage efficiency with moderate dielectric constant and moderate breakdown strength. 

**Figure 15:**
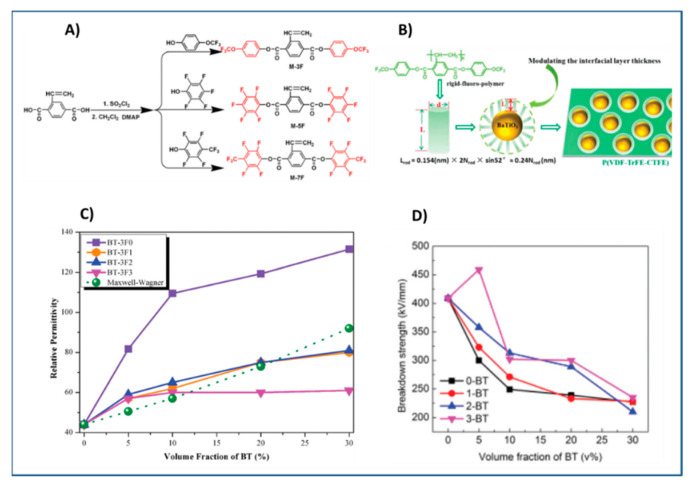
(**A**) Synthetic route for formation of TFMPCS monomer (**B**) schematic illustration of rigid-fluoropolymer@ BaTiO_3_/PVDF-TrFE-CTFE dielectric nanocomposite films (**C**) The permittivity of PVDF-TrFE-CTFE nanocomposites films with BT-3F0, BT-3F1, BT-3F2, and BT-3F3 at 1 kHz. (**D**) Variation of characteristic breakdown strength from Weibull distribution for samples with various volume fractions of fillers. Reproduced with permission from Ref. [130].

Some studies have systematically studied the role of pendant groups in the polymeric shell of encapsulated nanoparticles on the dielectric properties of nanocomposite. For example, Zhang and workers [170] varied the number of fluorine substituents present on the molecular structure of polymer shell of core shell structured rigid-fluoro-polymer@ BaTiO_3_ nanoparticles by performing RAFT polymerization with styrenic monomers containing different number of fluorine (M-3F, M-5F and M-7F) (Figure 15A), Evaluation of dielectric performance of the nanocomposites of rigid-fluoro-polymer nanoparticles@ BaTiO_3_ (P-3F, P-5F and P-7F) and poly (Vinylidene fluoride-trifluororethylene-chlorotrifluoroethylene (PVDF-TrFE-CTFE) indicated a strong dependance of permittivity and energy densities on the molecular structure of fluorinated styrenic monomer. For 5% loading, the nanocomposite formulated with fluorinated styrenic monomer containing 3F exhibited the highest breakdown strength (542 kV mm^−1^) and highest energy density (14.5 J cm^−3^) which could be attributed to compact interfacial interactions of P-3F with PVDF-TrFE-CTFE matrix.

Other studies have explored the effect of shell thickness on the dielectric properties of nanocomposite while maintaining similar composition of the polymer shell of core-shell nanoparticles. Zhang et al. [130] synthesized core shell structured rigid-fluoro-polymer@BaTiO_3_ nanoparticles via SI-RAFT polymerization of 2,5-bis[(4-trifluoromethoxyphenyl)oxycarbonyl] styrene (TFMPCS) with RAFT agent anchored to BaTiO_3_ nanoparticles. TFMPCS was synthesized starting from 2-vinylterephthalic acid as depicted in Figure 15A. The PGNPs were incorporated in PVDF-TrFE-CTFE matrix to study the dielectric properties of the nanocomposite. A careful analysis of the results revealed that the dielectric constant, breakdown strength and energy density of the polymer nanocomposites were significantly affected by the thickness of rigid-fluoro-polymer shell around the BaTiO_3_ nanoparticles. For instance, nanocomposite with higher shell thickness (i.e., obtained from BT-3F3) exhibited higher breakdown strength while dielectric permittivity showed an inverse relationship with shell thickness (Figure 15). This is expected because polymers in general have higher breakdown strength while bare nanoparticles have higher permittivity. The energy density for 5 vol% BT-3F3/PVDF-TrFE-CTFE nanocomposite (36.6 J cm^−3^ at the electric field of 514 kV mm^−1^) was significantly higher compared to pure PVDF-TrFE-CTFE (15.4 J cm^−3^ at the electric field of 457 kV mm^−1^). 

Similarly, Yang et al. [176] studied the effect of shell thickness of PGNPs on the dielectric properties of nanocomposite while keeping the polymer composition of core-shell NPs constant. The RAFT agent (EDMAT) was initially immobilized on the surface of silanized BaTiO_3_ nanoparticles by conducting reaction with n-hydroxysuccinimide activated ester of EDMAT (NHS-EDMAT). A series of PS @BaTiO_3_ nanoparticles were prepared by RAFT polymerization where, the shell thickness was tuned by changing the feed ratios of styrene and BaTiO_3_-EDMAT. The dielectric constant of single component core-shell (shell thickness varying from 7 to 12 nm) nanocomposite ranged from 14–24 depending upon the shell thickness (7 to 12 nm) and the dielectric loss ranged from 0.009–0.13. Additionally, the dielectric constant as well as the dielectric loss of all the nanocomposites showed a weak frequency dependence over a wider range of frequencies (1 Hz to 1 MHz). 

BaTiO_3_-EDMAT nanoparticles have not only been utilized for surface-initiated RAFT polymerization of styrene but also for the polymerization of fluoroalkyl acrylates viz., 1H,1H,2H,2H-heptadecafluorodecyl acrylate (HFDA) and trifluoroethyl acrylate (TFEA) [175]. The surface energies of poly(fluoroalkyl acrylate)are generally lower than those of hydrogenated polymers e.g., PS. Several fluoroalkyl acrylate monomers with different structures were grafted on BaTiO_3_ nanoparticles and surface-initiated RAFT polymerization was conducted so as to synthesize polymer grafted BaTiO_3_ nanoparticles with the least surface energy. Dielectric evaluation of fluoro-polymer@BaTiO_3/_PVDF-HFP nanocomposites revealed that the energy density of 50% PTFEA@BaTiO_3_/PVDF-HFP/nanocomposites (6.23 J.cm^−3^) was 150% greater than that of the pure PVDF-HFP (~4.10 J.cm^−3^). Further, nanocomposite derived from PTFEA@BaTiO_3_ exhibited slightly better dielectric performance over that derived from poly(1H,1H,2H,2H-heptadecafluorodecyl acrylate) PHFDA@BaTiO_3_ because PTFEA has pendent trifluoroethyl group which promotes a more compact interface compared to PHFDA which has long perfluoroalkyl pendant group.

The structure-property relationship study of polymer-grafted BaTiO_3_ nanoparticles (synthesized by RAFT technique) filled polymer nanocomposites clearly indicates that several factors influence the dielectric performance of the nanocomposite including the polymer composition of the shell, the pendant groups of the polymer shell, and the shell thickness of the core-shell nanoparticles.

## 5. SI-Nitroxide-Mediated Polymerization (SI-NMP) to Prepare Polymer Grafted Nanoparticles

Nitroxide-mediated polymerization (NMP) involves reversible activation−deactivation of propagating polymer chains by a nitroxide radical [198]. NMP polymerization has been widely used for grafting styrenic monomers however, other monomers methyl methacrylate, *n*-butyl acrylate, N-isopropylacrylamide, acrylic acid, etc. have also been grafted on the surface of NPs [32,199,200,201,202,203,204,205]. The initiators used for NMP polymerization include 2,2,6,6-tetramethylpiperidinyloxy (TEMPO) [57], N-tert-butyl-N-[1-diethylphosphono-(2,2-dimethylpropyl)] nitroxide (DEPN) [206] and TIPNO [207]. The, SI-NMP of grafting polymer chains on NPs involves initially immobilizing TEMPO or DEPN or TIPNO initiator functionalities on the NP surface [48]. The beauty of NMP polymerization is in that the nitroxide radical endcaps the polymer chain to form a persistent radical effect without the need for a separate initiator or catalyst (The propagating species are formed via dissociation of a nitroxide radical). During polymerization, the equilibrium between dormant and active species shifts towards the dormant species and limits the number of active radical species present and also restrict possible termination reactions. 

### 5.1. SI-NMP Polymerization to Prepare Polymer Grafted SiO_2_ Nanoparticles

**Figure 16:**
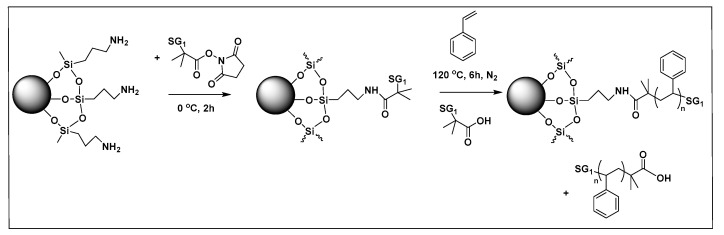
Scheme for anchoring of the MAMA-SG1 initiator and SI-NMP polymerization of styrene on the SiO_2_ NP surface. Reproduced with permission from Ref. [32].

Yang et al. [208] utilized SI-NMP polymerization for the preparation of polystyrene grafted SiO_2_ nanoparticles. The SiO_2_ nanoparticles were initially treated with thionyl chloride, and the modified nanoparticles were then reacted with tertiary butyl hydroperoxide (TBHP) to introduce peroxide groups on the surfaces of nanoparticles. Then NMP polymerization was initiated in the presence of TEMPO agent to graft polystyrene on the surface of SiO_2_ particles [208]. Alternatively, Chevigny and coworkers employed APTMS modified SiO_2_ nanoparticles and grafted MAMA-SG1 (BlocBuilder), (NMP initiator) for subsequent SI-NMP grafting of PS to SiO_2_ nanoparticles. The SI-NMP polymerization of styrene was carried out in the presence of free MAMA-SG1 as a sacrificial initiator to ensure a better control of the polymerization (Figure 16).

### 5.2. SI-NMP Polymerization to Prepare Polymer Grafted TiO_2_ and BaTiO_3_ Nanoparticles

SI-NMP has also been employed for grafting of PS [209,210] and poly(4-chloromethyl styrene-*g*-4-vinylpyridine) (PCMSt-*g*-P4VP) [211] on TiO_2_ as well as poly(4-hydroxystyrene) (PVP) [212] and poly(styrene-co-maleic anhydride) (PSMA) copolymers [213] on BaTiO_3_ nanoparticles to obtain surface modified NPs. However, there has been no dielectric data reported of polymer grafted nanoparticles synthesized using SI-NMP technique. 

## 6. Grafting to Method to Prepare Polymer Grafted Nanoparticles

The grafting to method is based on the use of polymer chain with functional groups that is randomly distributed along the chain or attached at the end of the polymer chain. The attachment of the graft polymer on nanoparticle surface requires coupling reaction of the functionalized backbone or the end-group functionalized polymer chain with the surface functionalized nanoparticles. Common reaction techniques used to synthesize functional polymers for grafting to method include free-radical polymerization, anionic polymerization, ATRP, and RAFT. The coupling reactions generally used in grafting to methods are click reactions, silanization, phophonate coupling, esterification, etherfication, etc. 

### 6.1. Grafting to Method to Prepare Polymer-Grafted SiO_2_ Nanoparticles

The combination of CRP techniques (ATRP, [214] RAFT, [215] etc.) and coupling reactions has been useful in grafting PMMA, PS, PNiPAAm, poly(N-vinylcarbazole), poly(7-(6-(acryloyloxy) hexyloxy) coumarin), etc. on SiO_2_ nanoparticles. Initially, silane-terminated polymer or phosphonate-terminated polymer is synthesized so as to graft polymer on nanoparticles [216,217,218]. For example, PS samples with end-functionalized dimethylchlorosilane of different molecular weights (8 kDa, 26 kDa, 108 kDa, and 126 kDa) were grafted on SiO_2_ bilayer. The bilayer was then used as organic-oxide hybrid gate dielectrics to fabricate solution-processed triethylsilylethynyl anthradithiophene (TES-ADT) organic field-effect transistors (OFETs). The molecular weights of PS chains significantly altered the areal grafting densities (due to steric hindrance), the interfacial structure and the dielectric properties as well as the performance of the OFETs. The lower molecular weight PS-*g*-SiO_2_ surface exhibited smoother brush like structure while higher molecular weight PS-*g*-SiO_2_ surface exhibited pancake like structure. The smoother surface of 8 kDa PS-*g*-SiO_2_ surface showed the highest mobility (2.12 cm^2^·V^−1^·s^−1^) whereas the pancake surface of 135 kDa PS-*g*-SiO_2_ showed the lowest mobility (0.85 cm^2^·V^−1^·s^−1^) [219]. 

### 6.2. Grafting to Method to Prepare Polymer Grafted TiO_2_ Nanoparticles

**Figure 17:**
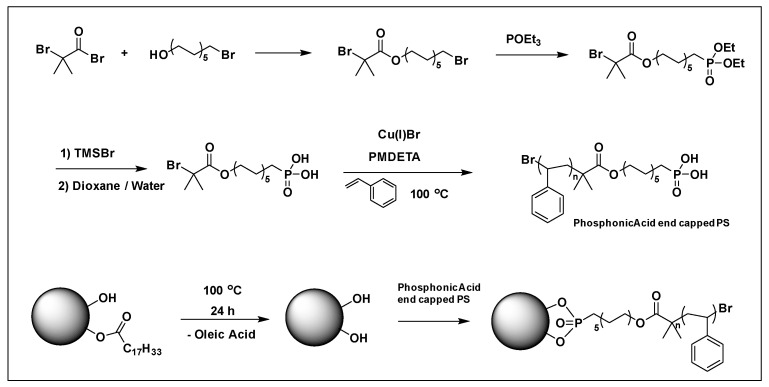
Synthesis of phosphonate end capped polystyrene and ligand exchange reaction of diethyl phosphonate end capped polystyrene with oleic acid terminated TiO_2_ nanoparticles to generate polystyrene coated TiO_2_ (PS@TiO_2_) Reproduced with permission from Ref. [220].

Phosphonic ester end capped PS has been synthesized using ATRP technique (Figure 17). The phosphonic acid end-functionalized PS was then coupled with oleic acid stabilized cylindrical shaped titanium oxide nanoparticles (TiO_2_-OLEIC) to obtain PS@TiO_2_. The PS@TiO_2_ nanoparticles thus prepared were used for the fabrication of capacitors as well as pentacene thin film transistors. The dielectric constant of single component PS@TiO_2_ nanocomposite was ~9 (which is nearly 3.6 times higher than that of polystyrene) at 18.2 volume % loading of PS @TiO_2_, while the mobilities of PS@TiO_2_/ITO (bilayer) approached 0.2 cm^2^/V·s. [220] showing the importance of synthesized PS grafted TiO_2_ nanoparticles via grafting to approach in electronics and dielectric applications. 

**Figure 18:**
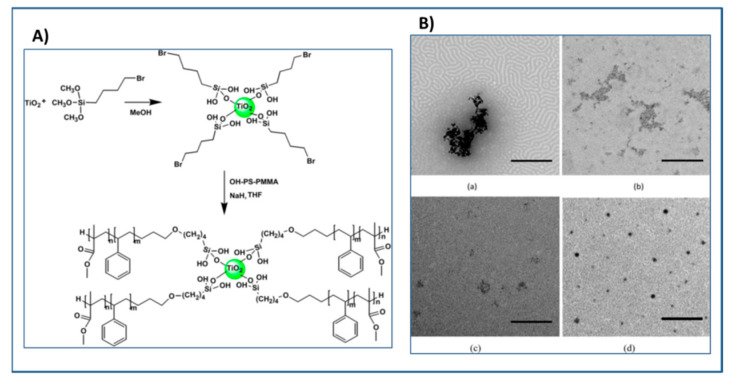
(**A**) Scheme for synthesis of PMMA-b-PS -*g*-TiO_2_ nanoparticles; (**B**) TEM (no stain) images of (**a**) oven annealed pristine TiO_2_ (10 wt%)-BCP (57 k-b-25 k) composite (**b**) As-cast PS (60 k) with 10 wt% BCP-*g*-TiO_2_ (**c**) As-cast PMMA (33 k) with 10 wt% BCP-*g*-TiO_2_, and (**d**) Oven annealed PMMA (33 k) with 10 wt% BCP-*g*-TiO_2_. (scale bar: 0.5 µm). Reproduced with permission from Ref. [221].

Using “grafting to” approach, block copolymer has been grafted to TiO_2_ nanoparticles with hydroxyl group as the end functionality of the anchoring block copolymer. Hailu and coworkers [221] demonstrated using “grafting-to” approach the ability to graft PMMA-b-PS-OH to silylated TiO_2_ nanoparticles to form block copolymer grafted nanoparticle (Figure 18). It was observed that the dispersion of PMMA-b-PS-*g*-TiO_2_ nanoparticles in PMMA and PS-PMMA BCP films was far better compared to that in PS films which could be attributed to the improved interactions of the outer corona of the PMMA-b-PS-*g*-TiO_2_ NPs with the PMMA component of BCP. The addition of 2.6 vol% of BCP-*g*-TiO_2_ NPs resulted in 18% enhancement in the permittivity and lower dielectric loss compared to the bare TiO_2_ nanoparticles filled BCP nanocomposite [222]. 

Similarly, using “grafting to” approach, block copolymer was grafted onto TiO_2_ nanoparticles with dopamine as the anchoring group. Obata et al. synthesized copolymer containing dopamine as pedant groups via RAFT technique and subsequently coupled it with TiO_2_ nanoparticles to yield block copolymer grafted TiO_2_ nanoparticles [223]. Alternatively, silylation approach can also be used to graft PMMA on TiO_2_ nanoparticles by coupling of TiO_2_ with preformed trimethoxysilyl functionalized PMMA that was synthesized via ATRP technique [224].

### 6.3. Grafting to Method to Prepare Polymer Grafted BaTiO_3_ Nanoparticles

The silylation route has also been used in the grafting to approach of polymer grafted BaTiO_3_ nanoparticles. Xie et al. [133] formed PVDF-HFP@BaTiO_3_ nanocomposites by initially synthesizing) P(VDF-HFP) with glycidyl methacrylate (GMA) functionality via ATRP (Fluorine atom of the PVDF-HFP was utilized to initiate the ATRP of the GMA) technique. The functionalized polymer was then reacted with the APTMS-functionalized BaTiO_3_ nanoparticles. The coupling reaction between PVDF-HFP-GMA (epoxy functionality) and amino-functionalized BaTiO_3_ yielded PVDF-HFP@BaTiO_3_ nanocomposite with superior dielectric properties. For example, the nanocomposite with 50% nanoparticle loading exhibited dielectric constant of 34.8 at 1 MHz, about 3.9 times greater than that of pristine PVDF-HFP while dielectric loss observed was 0.128 at 1 MHz.

**Figure 19:**
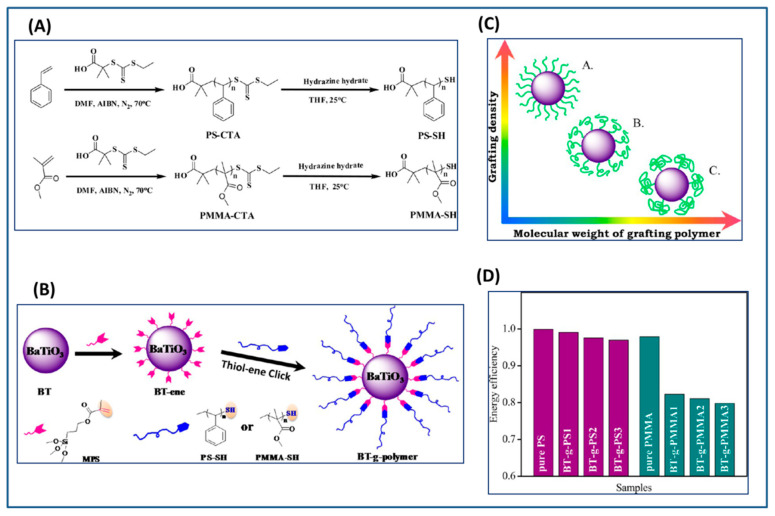
Schematic illustration for (**A**) synthesis of thiol-terminated PS and thiol-terminated PMMA via RAFT polymerization and (**B**) preparation of core-shell structured polymer@BaTiO_3_ nanocomposites by thiol−ene click reaction. (**C**) The relationship between the molecular weight of grafting polymer and grafting density of the core-shell structured nanoparticles (**D**) Energy efficiency of the core-shell structured polymer@BT nanocomposites under the electric field of 10 kV/mm. Reproduced with permission from Ref. [225].

Alternatively Yang and coworkers [225] synthesized core-shell structured polymer@BaTiO_3_ nanoparticles for dielectric applications via “grafting to” route using a combination of “thiol-ene” and silylation chemistry. Thiol-terminated PS or PMMA (molecular weight of PS1 and PMMA1~10K, PS2 and PMMA2~40K and PS3 and PMMA3~80K) were prepared by RAFT polymerization and was allowed to react with vinyl-functionalized (methacryloxypropyltrimethoxy) silanized BaTiO_3_ nanoparticles to form a series of polymer grafted nanoparticles PS@BaTiO_3_ and PMMA@BaTiO_3_ (Figure 19). It was observed that the graft density decreased with increase in the molecular weight of the grafted polymer. The dielectric constant of PS@BT (k = 30–33) and PMMA@BT (k = 34–38) single component nanocomposites was greater than that of pure polymers (k for PS = 2.74 and k for PMMA = 3.69) while the low dielectric loss (for PS@ BT = 0.013 and for PMMA@ BT = 0.032) was maintained over a wider range of frequency. Compared to PMMA@BT nanocomposites, PS@BT nanocomposites exhibited higher energy efficiency due to lower remnant polarization. Furthermore, the energy efficiency of both PS@BT and PMMA@BT nanocomposites exhibited a strong dependence on the molecular weight of the grafted polymer chains and the grafting density indicating that the design of core-shell nanoparticle filled polymer nanocomposites with high energy density and high energy efficiency is intricately related to the shell structure.

**Figure 20:**
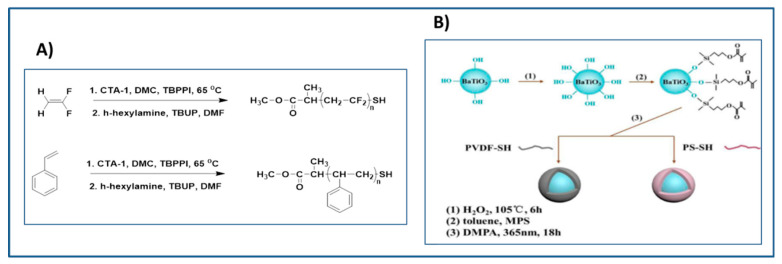
(**A**) Synthesis of SH-terminated PS and SH-terminated PVDF. (**B**) Grafting to approach for synthesis of hybrid nanoparticles. Reproduced with permission from Ref. [131].

Similarly, Ma et al. [131] synthesized core-shell structured PVDF@BT and PS@BT nanoparticles via thiol-ene coupling. Where thiol-terminated poly(vinylidene fluoride) (PVDF-SH) and thiol-terminated polystyrene (PS-SH), was reacted with γ-methacryloxypropyltrimethoxysilane (MPS) functionalized BaTiO_3_ nanoparticles (as depicted in Figure 20). It was observed that the dielectric permittivity and Eb of PVDF@BT/PVDF (117 kV/mm) and PS@BT/PVDF composites (107 kV/mm) was better than that of unmodified-BT/PVDF composites (58.5 kV/mm). The superior εr of PVDF compared to PS resulted in higher dielectric constant of PVDF@BT/PVDF (εr = 33 at 30% loading) over PS@BT/PVDF composites (εr = 25 at 30% loading). Furthermore, PVDF@BT NPs exhibited better compatibility with PVDF matrix compared to PS@BT resulting in improved breakdown strength of nanocomposite. In other words, PVDF shell act as a buffer layer and reduced the electrical mismatch between the matrix and core nanofillers compared to the PS hell. 

### 6.4. Grafting to Method to Prepare Polymer Grafted Al_2_O_3_ Nanoparticles

The “grafting to” techniques has also been used to graft PS with -COOH end groups by reacting with –OH groups on the surface of Al_2_O_3_ nanoparticles (Al NPs) [139]. The PS with -COOH end group was initially synthesized by free radical polymerization initiated by 4,4′ -azobis (4-cyanovaleric acid) (ACVA) in toluene. The grafting of high surface energy Al NPs with PS having -COOH end group greatly reduced the aggregation of Al NPs in comparison to the bare Al NPs in PS matrix. When the Al NPs and PS grafted Al NPs were mixed with PS to form PS nanocomposite films, the results showed larger voids for *agg*-Al NPs filled PS composite film but a more homogeneous composite film for PS grafted Al NPs. The dielectric constant of the pristine PS film, the PS films doped with 30 wt% *agg*-Al NPs and PS grafted Al NPs at 10^5^ Hz were found to be 2.80, 4.75 and 9.50, respectively. The breakdown strength and energy density of the PS film doped with PS grafted Al NPs (211–175 kVmm^−1^ and 1.70 J/cm^3^ at 1000 Hz) was greater than PS film doped with *agg*-Al NPs (183.77 to 30 kVmm^−1^ and 0.26 J/cm^3^ at 1000 Hz) and this was ascribed to the good compatibility and good dispersion of the PS grafted Al NPs in the PS film. 

PS-*g*-Al_2_O_3_ nanoparticles were also synthesized by silanization of Al_2_O_3_ NPs with dimethylchlorosilane-end-capped polystyrene (PS) to obtain grafted nanoparticles with graft density of 0.13 chains/nm^2^. The different wt% of PS-Al_2_O_3_ nanoparticles were blended with PS to fabricate nanocomposites with dielectric constant in the range 2.59 to 7.79. The nanocomposite film was found to be an efficient surface passivator for the oxide dielectric layer in organic field-effect transistors (OFETs). The field-effect mobility (1.4 × 10^–3^ cm^2^/V·s) and threshold voltage (4.4 V) of OFETs with PS-Al_2_O_3_ nanoparticles were found to be significantly better than that of nanocomposite with bare Al_2_O_3_ nanoparticles (field-effect mobility = 1.7 × 10^–4^ cm^2^/V·s threshold voltage = 6.7 V) [132]. 

## 7. In situ Polymerization to Prepare Polymer Grafted Nanoparticles

In situ polymerization has been widely used for producing well-dispersed metal oxide nanoparticle/polymer composite. In this technique, nano-sized metal oxide particles are mixed with organic monomers either in the presence or absence of a solvent followed by polymerization of the respective monomers. The nanoparticles are encapsulated in polymer shell via physical or chemical adsorption by taking advantage of the reactive functionality (e.g., acrylate functionality, it can be termed as “grafting through” or “grafting onto”) on NP surface. Both emulsion and suspension polymerization methods or in situ synthesis of both NPs (sol-gel synthesis) and polymers (free radical polymerization) have been employed [45,59,226,227,228,229,230]. 

Morales-Acosta and coworkers performed sol–gel and in situ polymerization using tetraethyl orthosilicate (TEOS) as SiO_2_ precursor, methyl methacrylate (MMA) as monomer, and 3-(trimethoxysilyl)propyl methacrylate (TMSPM) as coupling agent to improve the compatibility between PMMA and SiO_2_. Various core-shell nanoparticles with equimolar proportion of TEOS and MMA and varying concentrations of coupling agent, TMSPMA were prepared so as to study the effect of coupling agent concentration on the properties of fabricated nanocomposite films. All of the PMMA–SiO_2_ hybrid films exhibited higher dielectric constant (5.7 to 14) than that of PMMA (κ = 3.2 at 1 MHz) and bare SiO_2_ (κ = 3.9 at 1 MHz). The enhancement in the permittivity was attributed to residual solvents (-OH groups) and MMA (-C=C-groups, due to incomplete conversion into PMMA) present in the nanocomposite films [230]. 

Morales-Acosta and coworkers utilized low-temperature sol-gel and in situ polymerization to obtain PS- or PMMA-grafted-metal oxide (SiO_2_, TiO_2_, ZrO_2_) hybrid films for gate dielectric applications in the thin film transistors [231,232,233,234]. Similarly, Sánchez-Ahumada et al. synthesized PS-TiO_2_ hybrid dielectric films by performing sol-gel process with titanium butoxide (TB) as precursor and in situ polymerization of styrene in presence of the coupling agent, 3-trimetoxy-silyl-propyl-methacrylate (TMSPM) simultaneously. The dielectric constant of the hybrid film was 5.2 at 1 MHz, which is higher than that of pristine PS (2.74). PS-TiO_2_ hybrid dielectric films exhibited leakage current of 1 × 10^−6^ A/cm^2^ which is low enough to qualify the hybrid material as a dielectric gate in electronic devices [235]. 

PMMA embedded TiO_2_ nanoparticles were also synthesized via in situ free radical polymerization of methyl methacrylate using benzyl peroxide as an initiator in aqueous solution of polyvinyl alcohol (PVA) and sodium phosphate along with preformed TiO_2_ nanoparticles. The dielectric properties of PMMA embedded TiO_2_ nanoparticle filled PMMA nanocomposite showed high dielectric constant with low dielectric loss, [236].

Wang and coworkers reported the synthesis of PMMA-*g*-TiO_2_ via in situ emulsion polymerization technique. The dielectric study of PMMA-*g*-TiO_2_/PVDF-HFP nanocomposite film showed that the permittivity of the nanocomposite was enhanced by 13.9% compared to the pristine PVDF-HFP film whereas the breakdown field strength of the nanocomposite was nearly doubled compared to bare TiO_2/_PVDF-HFP nanocomposite. The enhanced dielectric performance of the nanocomposite resulted the improvement in the energy density of the PMMA-*g*-TiO_2_/PVDF-HFP nanocomposite (at 1 vol.% nanoparticle loading) by 14.4% w.r.t pristine PVDF-HFP (from 12.4 to 14.2 J/cm^3^) and an improvement in charge-discharge energy efficiency of 47% below 500 MV/m electric field [136].

Recently, Zhou et al. synthesized polyurea-grafted core-shell nanoparticles (PUA@BaTiO_3_) via in situ polymerization using 4,4′-methylene diphenyl diisocyanate and 4,4’-oxydianiline as monomers. The PUA@BaTiO_3_ nanoparticles were subsequently blended with PVDF-CTFE to fabricate nanocomposite films for evaluation of dielectric properties. The incorporation of PUA@BaTiO_3_ in PVDF-CTFE matrix resulted in 1.65 times higher energy density (8.94 J/cm^3^) than that of pristine PVDF-CTFE (5.41 J/cm^3^). Further, the energy density of PUA@ BaTiO_3_/PVDF-CTFE nanocomposite was also 1.45 times higher than that of pristine BaTiO_3_/PVDF-CTFE nanocomposite [237]. 

Similarly, Jinhong et al., [141] reported the grafting of hyperbranched aromatic polyamide on Al_2_O_3_ nanoparticles (HBP@Al_2_O_3_) and the use of functionalized nanoparticles to enhance the dielectric properties of epoxy nanocomposite. The incorporation of HBP@Al_2_O_3_ nanoparticles to epoxy matrix resulted in an enhancement in the glass transition temperatures (176.3 to 208.1 °C with 20 wt% of the filler). Furthermore, the dielectric constant of the HBP@Al_2_O_3_/epoxy nanocomposite was reported to be 5.0, compared to neat epoxy (3.5) and that of composite formed with 20 wt% bare Al_2_O_3_ nanoparticles (4.75). It was concluded that the improvement of Tgs (176.3 to 208.1 °C), dielectric strength (29.40 to 32.83 KV/mm) and the reduction of dielectric loss (0.024 to 0.020) were due to the good dispersion of the grafted NPs in the polymer matrix and also because of good interfacial adhesion of the grafted hyperbranched aromatic polyamide Al_2_O_3_ nanoparticles with the epoxy matrix.

## 8. Templated Approach to Prepare Polymer Grafted Nanoparticles

Template-assisted method involves the formation of nanoparticles within the specific area of the template and the method can be efficiently used for fabrication of well-defined core-shell nanomaterials. Especially, template-assisted polymer grafting approach has been employed to control the size and shape (spherical, cylindrical, nanotubes, etc.) of core nanostructure as well as the structure of graft present on the surface of the nanoparticles [58,238,239,240,241,242,243,244,245,246,247,248,249,250]. 

Template-assisted polymer grafting which offers an easier way to synthesize nanoparticles (in situ) through micelle formation is a relatively straightforward technique. However, Gou et al. noticed bimodal distribution of PS/PMMA-*g*-CdS quantum dots on the core of the self-assembly of PS-*b*-PAA-b-PMMA triblock copolymer micelles [46]. This aspect was addressed by the selection of unimolecular star block copolymer micelles which often yields hairy nanoparticles with uniform sizes, various shapes, and sometimes unusual morphologies [47]. 

**Figure 21:**
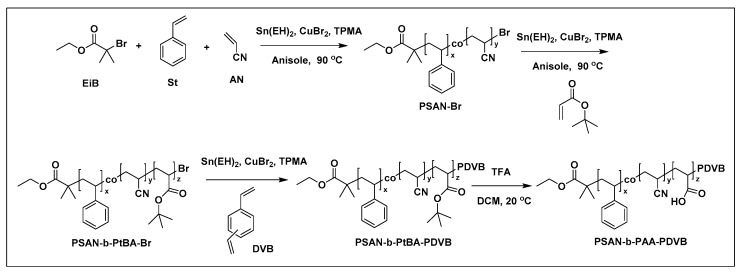
Synthesis of PSAN-*b*-PAA-PDVB star-shaped polymer templates. Reproduced with permission from Ref. [252].

Matyjaszewski and coworkers demonstrated the utilization of poly(styrene-co-acrylonitrile)-b-poly (acrylic acid)-poly(divinylbenzene) (PSAN-*b*-PAA-PDVB) star-shaped copolymers obtained via activator regenerated by electron transfer atom transfer radical polymerization (ARGET ATRP) (as depicted in Figure 21) as template for the synthesis of TiO_2_ nanoparticles. PMMA gate dielectrics layers fabricated with 0.4% wt. of the hybrid TiO_2_ nanoparticles was used in the measurement of organic field effect transistors (OFETs). The efficiency of OFETs was significantly better than OFETs based of pure PMMA as gate dielectric (charge carrier mobility has increased nearly 10-fold from ~0.06 to ~0.5 cm^2^/V·s). The improved performance of OFET could be ascribed to a significant decrease of roughness of dielectric layer (root mean square roughness was reduced from 15.3 nm to 0.43 nm) and changes to the surface energy (from 32.4 to 45.5 mN/m) of the gate dielectric layer after incorporation of hybrid nanoparticles [251]. 

Guo et al. synthesized PS-grafted BaTiO_3_ nanoparticles with sizes of 11 nm and 27 nm using amphiphilic star-like poly(acrylic acid)-*b*-polystyrene (PAA-*b*-PS) diblock copolymer templates. PAA-*b*-PS was obtained by sequential atom transfer radical polymerization [252,253]. The dielectric performance with respect to temperature was studied for PS-BaTiO_3_ nanoparticles of 11 nm and 27 nm. 

Lin and coworkers also prepared PS-functionalized BaTiO_3_ NPs with different sizes (~27 nm and ~11 nm) by exploiting amphiphilic unimolecular star-like PAA-*b*-PS diblock copolymer as template. The synthesized nanoparticles were dispersed in low molecular weight PS-b-PMMA (M_PS_ = 45,900 and M_PMMA_ = 138,000) and high molecular weight PS-b-PMMA (M_PS_ = 315,000 and M_PMMA_ = 785,000) to fabricate PS@BaTiO_3_/PS-*b*-PMMA nanocomposite thin film. The incorporation of PS@BaTiO_3_ NPs into PS-*b*-PMMA, resulted in the preferential location of the BaTiO_3_ NPs in the PS nanocylinders. PS grafting to BaTiO_3_ NPs not only prevented aggregation by van der Waals forces, but also offered selective chemical affinity to the PS block of BCP. The measurements of dielectric properties of nanocomposite thin film revealed that the dielectric performance of the film was dependent upon the molecular weight of PS-*b*-PMMA and the size of PS@BaTiO_3_ NPs. BCP nanocomposite of 27 nm PS@BaTiO_3_ NP exhibited higher permittivity than that of 11 nm PS@BaTiO_3_ NP due to higher dielectric constant of large sized 27 nm NPs. Moreover, it was noticed that the nanocomposites of low molecular weight BCP exhibit higher dielectric constant than that of nanocomposite of high molecular weight due to lower permittivity of high molecular weight BCP. The low permittivity of higher molecular weight polymers could be attributed to the higher degree of chain coiling of longer polymer grafts than the low molecular weight polymer grafts [254]. 

**Figure 22:**
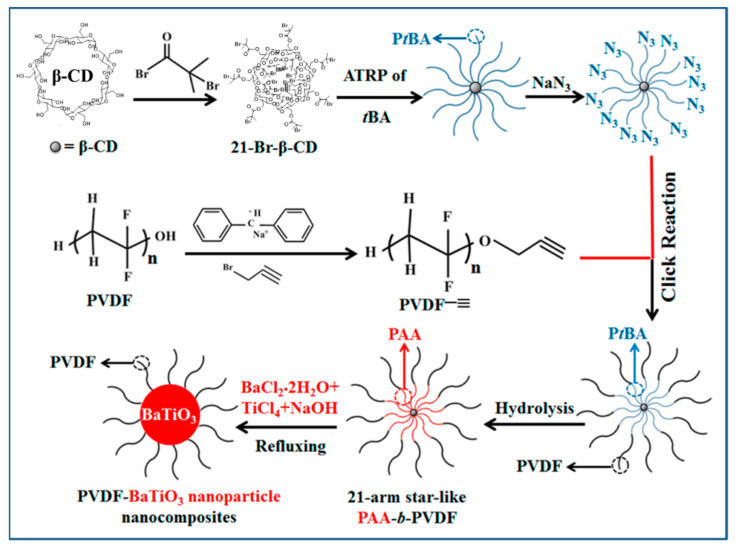
Synthesis of amphiphilic 21-arm, star-like PAA-*b*-PVDF diblock copolymer and PVDF@BaTiO_3_ nanoparticles. Reproduced with permission from Ref. [58].

Jiang and coworkers [58] synthesized PVDF-functionalized BaTiO_3_ nanoparticles by template-assisted approach. Firstly, they synthesized amphiphilic star diblock copolymer, by ATRP technique (Figure 22). PAA-*b*-PVDF (PAA as inner hydrophilic block while PVDF as outer hydrophobic block with well-controlled molecular weight of narrow dispersity) was dissolved in a mixture of DMF and benzyl alcohol followed by the addition of BaCl_2_.2H_2_O and TiCl_4_ as precursor and NaOH. Precursors assemble in the space of PAA blocks and PVDF chains serve as the arm of the self-assembled structure. The size of the nanoparticles was tuned based on the molecular weight of the PAA and PVDF blocks of the star copolymer. Notably, PVDF-BaTiO_3_ nanocomposites (single component) displayed not only high dielectric constant (~80 at 100 Hz) but also low dielectric loss (<0.2) over broad frequency range as compared to PVDF.

However, one of the challenges of unimolecular star block copolymer micelles using template approach in formulating core shell nanoparticles is the low graft density of the polymer grafted nanoparticles. Like the grafting to method, template assisted polymer grafting is not as widely sought-after technique for the synthesis of core-shell nanoparticles.

## 9. Discussion

Current technologies for pulsed power applications utilize polymers as the dielectric material of choice due to their high electrical resistance, low dielectric loss, self-healing capability, formability and flexibility. However, the most widely used polymeric system namely metallized biaxially oriented polypropylene (BOPP) and its variants do not meet the demands of next generation film dielectrics. Composite dielectrics offer an unique opportunity to combine the high ε_r_ of inorganic fillers with the high E_bd_ of a polymer matrix to achieve high energy density capacitors. For significant gains in permittivity in polymer composites so as to achieve higher energy density, loadings of dispersed fillers in the composite need to be above 20% *v*/*v*. At these loading levels, achieving good NP dispersion—especially in non-polar polymer matrices—is challenging due to particle agglomeration during film preparation. Aggregated nanoparticles of high permittivity act as electrical field expulsion defect centers in filled polymers. Such defect centers effectively distort the distribution of electric field, making the local electrical field in the matrix much higher than the average electric field and also lower the overall energy storage of the nanocomposite. The extent of the field distortion is adversely influenced by the discontinuous (sharp and large) permittivity contrast between the NPs and the polymer matrix. An approach to address the field distortion is to consider high permittivity nanoparticles with core-shell architectures so that the nanoparticles permittivity gradually approaches that of the polymer matrix. 

We discuss various approaches to synthesize polymer grafted nanoparticles. Among the three commonly used SI-CRPs, SI-ATRP has been shown to be one of the most versatile polymerization techniques because it can be used under broad experimental conditions and can be adapted to synthesize nanoparticles with polymer grafts having a wide range of functional groups. Additionally, ATRP can be used to synthesize core@ double-shell structured nanoparticle via a two-step process. It was noted that the thickness of the second shell can be controlled by adjusting the ratio of monomer and macro initiator. The polymerization of activating monomer by ATRP process requires the use of alkyl halide initiator and a transition metal complex as catalyst (e.g., CuBr/ligand). However, the persistence of small amount of copper catalyst in the grafted nanoparticle can pose challenges because of the potential adverse effect the copper ions could have on the dielectric properties of the nanocomposite. In this regard, ATRP techniques with extremely low amounts of copper have been investigated for the synthesis of polymer grafted nanoparticles. For example, PS and PMMA were grafted from phosphonic acid functionalized BaTiO_3_ NPs via activated regenerated by electron transfer (AGRET) ATRP approach using only ppm amount of the copper catalyst [27]. Alternatively, efforts have focused on conducting ATRP using light without the addition of any metal catalyst and the thickness of polymeric shell was tunable based on the duration of white LED irradiation. These approaches to conduct ARGET ATRP with limited Cu species or light mediated ATRP offer novel opportunities and new routes for engineering surfaces and interfaces of nanoparticles with polymer grafts without significant copper residues. 

In contrast to ATRP, RAFT can be considered as a conventional radical polymerization with the addition of a chain transfer agent (CTA), which mediates the polymerization. The RAFT CTAs can be bound on nanoparticle surfaces via two main approaches. In the first approach, the CTA is synthesized with a reactive anchoring group (chlorosilyl group, phosphonic acid group) and then covalently bound to an unmodified NP. The second approach relies on grafting a functional RAFT agent to pre-modified nanoparticles. There are several examples in the literature where RAFT polymerization has been successfully used to synthesize unimodal or bimodal brush modified nanoparticles so as to achieve optimum dielectric performance of nanocomposite [177,178,186,187]. Using RAFT technique, the shell thickness, polymer composition and the aerial density of polymer brush in the core-shell nanoparticles have been successfully tuned and used for energy storage applications.

Despite SI-ATRP being the most predominant and sought after technique for polymer grafting on nanoparticle surface, RAFT technique too has steadily gained popularity over the years. This is because of the adaptability of RAFT to a range of polymerization conditions. Further advances in RAFT polymerization include the ability to synthesize multi-block copolymers with a high degree of fidelity, conducting reactions in the presence of oxygen and its compatibility with a broad range of functional groups and the absence of copper residues after polymer grafting have clearly contributed to the recent drive for use of RAFT technique in the synthesis of core-shell nanoparticles. 

The “grafting to” approach, on the other hand, involves surface modification of nanomaterials with functionality which is complimentary to the end-group functionality of polymer followed by coupling via suitable conjugation or click chemistry. In particular, chloro silyl-terminated polymer or phosphonate-terminated polymer or click chemistry has been used to graft polymer on nanoparticles. Because of the ability to precisely control the molecular weight of grafts in the grafting to technique, a study of low molecular weight grafts on NP revealed a relatively smooth surface while high molecular weight grafts on NP revealed a pancake like structure suggesting the ability of grafting to technique to control microstructure of the polymer shell at the expense of polymer graft molecular weight. Although ‘‘grafting-to” approach is easy and efficient, it presents challenges such as decreased graft density with increase in the molecular weight of the grafted polymer. Therefore, grafting to technique is not as widely sought after method for the synthesis of core-shell nanoparticles.

Template-assisted polymer grafting which offers an easier way to synthesize nanoparticles (in situ) through micelle formation is a relatively straightforward technique. An issue that has been observed is the formation of multiple NPs in the core of multi-molecular micelles [46]. To overcome this challenge, the use of unimolecular star block copolymer micelles has been tried out with a greater success in the synthesis of hairy nanoparticles with uniform sizes, various shapes, and sometimes unusual morphologies [47]. However, one of the challenges of unimolecular star block copolymer micelles using template approach is the low graft density of the polymer grafted nanoparticles. Like the grafting to method, template assisted polymer grafting is not as widely sought after graft technique for the synthesis of core-shell nanoparticles.

In in situ polymerization method, metal oxide nanoparticles are usually mixed with organic monomers, either in the presence or absence of a solvent, and then the monomers are polymerized. There is a thermodynamic compatibility at the polymer matrix-nanoparticle reinforcement interface and thus provide stronger matrix dispersion bond with very good miscibility of the nanocomposite. 

Several structure-property relationship studies of polymer grafted nanoparticles filled polymer nanocomposites [27,119,120,121,124,125,126] using SI-CRP technique have been conducted and they clearly indicate that a number of factors influence the dielectric performance of the nanocomposite including the thickness of the core and the shell of the core-shell nanoparticles and the type of polymer grafted on the nanoparticles, interfacial separation between core NPs and polymer shell, the composition of nanocomposite (single or multicomponent type of nanocomposite), the presence of polarizable interfacial layer and double shell coverage of nanoparticles. For establishing clear structure-property relationships, an efficient initiator and control of polymer brush graft density is important. At present, the techniques for facile determination of polymer brush grafting density and the initiator efficiency are scarce. More importantly, a simple, versatile and accurate technique for determining the number of initiator units per square nanometer present on the nanoparticle surface is not available. This information would be highly relevant for the development of composition-structure-property relationship paradigm of single or multi component nanocomposite.

Another interesting application of core-shell structure is in the pursuit of all-polymer field-effect transistors in the generation of polymer brush-based gate dielectrics. Especially SI-CRP technique has drawn significant attention for design of polymer field effect transistors. The attractiveness in the use of SI-CRP technique is in the ease of device preparation and the avoidance of expensive fabrication facilities. The performance of pentacene-based thin-film transistor fabricated from PS-grafted SiO_2_ and PMMA-grafted SiO_2_ using ATRP technique as a gate dielectric showed the importance of interfacial material and its structure in the design of OFET [97,98]. The device fabricated from 47 nm thickness of PS brush exhibited highest mobility (µ_FET_ = 0.099 cm^2^/V·s) indicating that optimum molecular weight polymer brushes need to be grown from surface of dielectric for achieving desirable performance. On the other hand, the surface-grafted PMMA brush (10 nm)/SiO_2_ (9 nm) on silicon wafer exhibited lower leakage than that of surface-grafted PMMA brush (20 nm) on silicon wafer (free of 9 nm SiO_2_ layer). Additionally, it was noted that the thickness of the polymer brush on silica could be modulated based on the activity of the catalyst, the reactant concentration and reaction time. The PMMA brushes on silica showed excellent insulating characteristics, large capacitance, and low charge-trapping density. Field-effect transistors with PMMA brush as the dielectric layer demonstrate excellent charge transport. However, the field of polymer brush-based hybrid materials in OFET is still in its infancy stage [43] and it needs further exploration.

## 10. Summary and Future Outlook

In this review article, we described various synthetic approaches for preparation of core-shell structures of polymer grafted nanoparticles. The grafting of polymeric chains to nanoparticles can generally be accomplished by four approaches namely (i) ‘grafting to’; (ii) ‘grafting from’; (iii) templated and (iv) in situ polymerization or encapsulation. All the four approaches yield polymer grafted nanoparticles of varying shell architectures. Unlike grafting to method, grafting from method allows to synthesize nanoparticles with high grafting density and polymer shells of varied composition. The “grafting from” method is also termed the surface-initiated controlled radical polymerization where the initiator functionality (SI-ATRP) or CTA functionality (SI-RAFT) or alkoxy amine functionality (NMP) is anchored to the surface of nanomaterials followed by growth of polymer chains. 

Recent progress in the various synthetic strategies for formulation of core-shell nanoparticles has created a myriad of architectures of core-shell nanoparticles. Interesting polymer architectures with unique features such as polymer loops, bottlebrushes have made the polymers synthesized by SI-CRP technique a valuable toolkit that can be used for a broad range of applications. Notably, the opportunity to synthesize BCP-*g*-NPs in formulating single-component hybrid materials has largely been unexplored and needs to be tapped for the design of nano-dielectrics. A high-performance core-shell hybrid material in which the polymer is directly grown from the nanoparticles provides an opportunity to synthesize single component nano-dielectrics. This subject need further exploration because it is possible to have high ceramic loading in the one component polymer-ceramic system with minimal negative effect of ceramics towards electrical discharge due to controlled minimal aggregation, i.e., high degree of dispersion. From an academic perspective, little is known about the structure and dynamics of self-assembling of single component BCP-*g*-NP system, i.e., whether a block copolymer tagged to nanoparticle can microphase-separate and self-assemble. There is also significant interest for developing molecular level understanding of non-centrosymmetric materials from fundamental perspective, and the parameter space it presents in terms of grafting density, and copolymer length and composition is wide open. Under the appropriate processing conditions, symmetric BCPs can microphase separate to form parallel lamellae [255,256,257]. This arrangement presents a unique opportunity to advance the field of core-shell nanoparticles in the formulating next generation nano-dielectrics of unprecedented performance.

## Figures and Tables

**Table 1:** Comparison of advantages and disadvantages of polymer grafting methods.

Grafting Methods	Advantages	Disadvantages
Grafting to	A number of coupling reactions and click reaction are available. Well-defined end-functionalized polymers can be obtained from CRPs. Clean approach, less labor intensive [33]	Due to the steric hindrance high grafting density could not be achieved. The approach is limited to polymer grafts with defined end groups. The surface of nanoparticles may have unreacted functionality
Grafting from	High grafting density, tuning of thickness with molecular weight of growing chain is possible [48]	The stringent reaction conditions have to be maintained.
Templated	Well-defined size of nanoparticles can be obtained [58]	Scalability is difficult. Not cost effective
In situ polymerizations	The technique is scalable and similar to conventional free radical polymerization [59]	Difficulty in controlling grafting density and molecular weights. Well defined structures such as block copolymers cannot be synthesized.

**Table 2:** Comparison of advantages and disadvantages of grafting from methods.

Grafting from Methods	Advantages	Disadvantages
ATRP	Control of molecular weights and dispersity. Variation to ATRP technique broaden the applicability of the technique to a range of surface initiated polymer grafting [48]	Small amount of copper persists along with polymer, its removal is difficult and affects the properties of the final product. Not suitable for acidic monomers Difficulty in synthesizing high molecular weight grafts [71]
RAFT	Adaptability of RAFT to a range of polymerization conditions high degree of fidelity, ability to work in the presence of oxygen, compatibility with a broad range of functional groups [48]	Because of the presence of sulfur containing moiety RAFT polymers are often colored and have foul odor and the synthesis of RAFT agents involves multiple steps [48]
NMP	NMP is one of the successfully used SI-CRP techniques for polymer grafting [72]	However, it is not applicable for most of monomers and functional groups [48] It requires high temperatures and longer time due to slow polymerization kinetics. There are difficulties associated with synthesis and stability of nitroxide and alkoxy amine [73]

**Table 3:** Summary of dielectric and electronic properties of polymer brushes grafted from SiO_2_ using ATRP.

Polymer Grafted Filler	Mean Diameter	Polymer Diameter/Graft Density	Active Semiconductor Layer	Molecular Weight	Capacitance (nF/cm^2^)	Eb (MV/cm)	*VT*	µ_FET_ cm^2^/(V·s)
PS-*g*-SiO_2_ (WF) [98]	300 nm	113 nm	Pentacene	135,000 g/mol	7.5 @ 100 Hz	NA	−38	0.094
PMMA-*g*-SiO_2_ [101]	~9 nm	~10 nm	Pentacene	NA	142 @ 1 MHz	7	−1	~0.2
PMMA-*g*-SiO_2_ [70]	2–3 nm	10 nm	CuPc	NA	220 @ 1 MHz	NA	−0.75	0.12

**Table 4:** Details about various polymer-grafted nanoparticles using ATRP.

Polymer-Grafted	Nanomaterial	Anchoring Moiety	Polymerization Conditions	Ref.
PPMA	BaTiO_3_	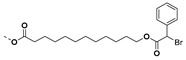	White Light, Photocatalyst5,10-di(1-naphthyl)-5,10-dihydrophenazine DMF, RT	[120]
Poly(2- hydroxyl ethyl methacrylate)-b-poly (methyl methacrylate); Sodium polyacrylate-b-poly(2-hydroxyl ethyl methacrylate)	BaTiO_3_	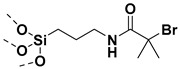	CuBr/CuBr_2_, PMDETA H_2_O/DMF, 60 °C, 24 h	[127]
Poly(1H,1H,2H,2H-perfluorooctyl methacrylate)	BaTiO_3_	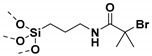	CuBr, PMDETA, DMF 70 °C, 24 h	[126]
PMMA	BaTiO_3_	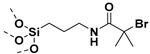	CuBr, PMDETA, DMF 60 °C, 24 h	[119]
PMMA Poly(Trifluoroethyl methacrylate) PTFEMA	BaTiO_3_	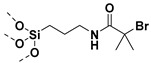	CuBr, PMDETA, DMF 70 °C, 12 h	[121]
PMMA	BaTiO_3_	BaTiO_3_@TMPc-Br	CuCl/CuCl_2_, Me_6_TREN, 60 °C, 24 h	[124]
PMMA	BaTiO_3_	BaTiO_3_@APS@HBP-Br	CuBr, PMDETA, 60 °C, 24 h	[125]
PS/PMMA	BaTiO_3_	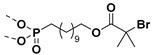	CuCl_2_, Me_6_TREN, Tin(II) ethylhexanoate, Anisol, 110 °C	[27]
Poly(lauryl methacrylate)	Al_2_O_3_	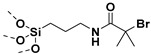	CuBr/CuBr_2_, HMTETA Toluene, 100 °C, 16 h	[128]

**Table 5:** Summary of dielectric properties of polymer nanocomposites fabricated from polymer brushes-grafted ceramic nanoparticles.

Polymer@filler	Mean Diameter	Shell Thickness (nm)	% Loading	Matrix	Grafting Approach	εr	tan δ	E_b_ (kV/mm)	Energy Density U (J/cm^3^)
PS@BaTiO_3_ [129]	~7 nm	NA	22% *v*/*v*	PS	Grafting to	5.8	NA	143	NA
PTFMPCS@BaTiO_3_ [130]	100 nm	11 nm	5 vol%	PVDF-TrFE-CTFE	SI-RAFT	~58	NA	459	36.6 @514 kV/mm
PVDF@BaTiO_3_ [131]	~100 nm	NA	30 vol%	PVDF	Grafting to	27.9	0.08872	117.3	NA
PS@Al_2_O_3_ [132]	50 nm	0.13	25 wt%	PS	Grafting to	2.63	NA	NA	NA
PS@Al_2_O_3_ [132]	50 nm	0.13	25 wt%	PMMA	Grafting to	3.19	NA	NA	NA
PS@BaTiO_3_ [131]	~100 nm	NA	30 vol%	PVDF	Grafting to	23.6	0.0866	107	NA
P(VDF-HFP)@BaTiO_3_ [133]	100 nm	NA	50 vol%	NA	Grafting to	34.8	0.128	20 MV/m	0.3 @20 MV/m
PGMA@BaTiO_3_ [134]	<100 nm	~20 nm	NA	PGMA	SI-ATRP	54	0.039	~3 MV/m	~21.51 @3 MV/m
PHEMA@PMMA @BaTiO_3_ [127]	100 nm	10 nm	38 vol%	NA	SI-ATRP	NA	~0.025	NA	~0.061 @70 kV/cm
PANa@PHEMA@BaTiO_3_ [127]	100 nm	10 nm	21 vol%	NA	SI-ATRP	NA	~0.022	NA	~0.09 @70 kV/cm
PMMA@ BaTiO_3_ [27]	50 nm	NA	22 vol%	NA	SI-ATRP	11.4	NA	218	3 @~220 V/μm
PTTEMA@BaTiO_3_ [135]	~50 nm	14–15 nm	20 vol%	PTTEMA	SI-RAFT	∼20	<0.02	~220	~3.4 @210 V/μm
PMMA@BaTiO_3_ [119]	100 nm	10 nm	76 wt%	NA	SI-ATRP	14.6	0.0372	NA	NA
PMMA@BaTiO_3_ [121]	~200 nm	7 nm	80 wt%	PVDF	SI-ATRP	~28.5	0.025 @100 kHz	NA	NA
PTFEMA@BaTiO_3_ [121]	~200 nm	4.5 nm	80 wt%	PVDF	SI-ATRP	~35	0.022	NA	NA
PPFOMA@BaTiO_3_ [126]	30–50 nm	5 nm	70.70 wt%	NA	SI-ATRP	7.4	0.01	NA	NA
PMMA@TiO_2_ [136]	50 to 100 nm	5 nm	1 vol%	PVDF-HFP	In situ	10.5	<0.04	560	14.2 @500 V/μm
PS@TiO_2_ [137]	40–50 nm	NA	27 wt%	NA	Grafting to	6.4	0.04	NA	NA
PS@TiO_2_ [129]	18 nm	NA	39% *v*/*v*	NA	Grafting to	12.8	0.1	114	NA
PS@TiO_2_ [138]	25–30 nm	NA	36.9 vol%	PS	SI-RAFT	~65	~0.03	NA	NA
PS@Al_2_O_3_ [139]	50–200 nm	0.12	30 wt%	*iso*-Al NPs@PS	Grafting to	9.50	0.01	175	1.70
PEB@Al_2_O_3_ [140]	100 nm	2–5 nm	25.0 vol%	PP	Grafting to	5.7	NA	37.5	NA
HBP@Al_2_O_3_ [141]	30 nm	NA	20 wt%	Epoxy	In situ	5.0	<0.025	32.83	NA
PP@Al_2_O_3_ [142]	140 nm	NA	10.4 vol%	NA	In situ	10.5	0.24	120	14.4 @120 V/μm

**Table 6:** Details about various polymer grafted nanoparticles using SI-RAFT.

Grafted Polymer	Nanoparticle	Anchoring CTA	Polymerization Conditions	Ref.
Poly(vinylidene fluoride)	BaTiO_3_	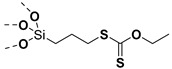	TBPPi, DMC 65 °C, 15 h	[169]
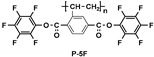	BaTiO_3_	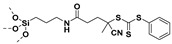	AIBN, THF 80 °C, 15 h	[170]
Poly{2,5-bis[(4-methoxyphenyl)oxycarbonyl]styrenes} (PMPCS)	BaTiO_3_	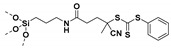	AIBN, THF 80 °C, 6 h	[171]
Polystyrene	BaTiO_3_	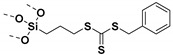	AIBN, DMF 80 °C, 12 h	[172]
Poly(2-(2,2′:5′,2″-terthien-5-yl)ethyl methacrylate) (PTTEMA)	BaTiO_3_	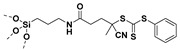	AIBN, Dioxane 90 °C, 3 h	[173,174]
Poly(1H,1H,2H,2H-heptadecafluorodecyl acrylate) (PHFDA)	BaTiO_3_	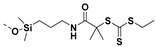	AIBN, DMF 60 °C, 6 h	[175]
Polystyrene	BaTiO_3_	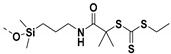	AIBN, DMF, 80 °C	[176]
Poly(stearyl methacrylate)	SiO_2_	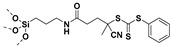	AIBN, THF, 60 °C	[177,178]
Poly(2-hydroxyethyl methacrylate)	SiO_2_	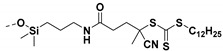	AIBN, THF, 70 °C	[164]
Poly(acrylic acid)	SiO_2_	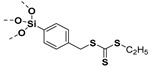	AIBN, DMF 70 °C, 3 h	[162]
Poly(stearyl methacrylate)	SiO_2_	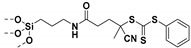	AIBN, THF, 60 °C	[177,178]
Polystyrene	TiO_2_	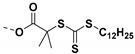	110 °C, 96 h	[138]
Poly(methyl methacrylate)-b-polystyrene	TiO_2_	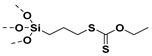	AIBN, DMF, 90 °C, 6 h	[179]

## Data Availability

No new data were created or analyzed in this study. Data sharing is not applicable to this article.

## Sample Availability

Samples of the compounds are not available from the authors.
